# Transcriptomic Analysis Reveals Competitive Growth Advantage of Non-pigmented *Serratia marcescens* Mutants

**DOI:** 10.3389/fmicb.2021.793202

**Published:** 2022-01-04

**Authors:** Tingting Xiang, Wei Zhou, Cailing Xu, Jing Xu, Rui Liu, Nuo Wang, Liang Xu, Yu Zhao, Minhui Luo, Xiaoxin Mo, Zeyang Mao, Yongji Wan

**Affiliations:** Laboratory of Invertebrate Pathology and Applied Microbiology, College of Sericulture, Textile and Biomass Sciences, Southwest University, Chongqing, China

**Keywords:** *Serratia marcescens*, spontaneous mutation, color morphotypes, growth advantages, transcriptomic analysis

## Abstract

*Serratia marcescens* is a common bacterium well-known for the red secondary metabolite prodigiosin. However, color mutants have long been described. Non-pigmented strains can be found to exist both naturally and under laboratory conditions. It is unclear why *S. marcescens* loses prodigiosin synthesis capacity in certain conditions. In the present study, we find that the spontaneous color mutants arise within a few generations (about five passages) and rapidly replace the wild-type parent cells (about 24 passages), which indicates a growth advantage of the former. Although, the loss of prodigiosin synthesis genes (*pigA-N*) is frequently reported as the major reason for pigment deficiency, it was unexpected that the whole gene cluster is completely preserved in the different color morphotypes. Comparative transcriptomic analysis indicates a dramatic variation at the transcriptional level. Most of the *pig* genes are significantly downregulated in the color morphotypes which directly lead to prodigiosin dyssynthesis. Besides, the transcriptional changes of several other genes have been noticed, of which transcriptional regulators, membrane proteins, and nearly all type VI secretion system (T6SS) components are generally downregulated, while both amino acid metabolite and transport systems are activated. In addition, we delete the transcription regulator *slyA* to generate a non-pigmented mutant. The Δ*slyA* strain loses prodigiosin synthesis capacity, but has a higher cell density, and surprisingly enhances the virulence as an entomopathogen. These data indicate that *S. marcescens* shuts down several high-cost systems and activates the amino acid degradation and transport pathways at the transcriptional level to obtain extra resources, which provides new insights into the competitive growth advantage of bacterial spontaneous color mutants.

## Introduction

*Serratia marcescens*, a bacterium of the *Enterobacteriaceae* family which produces a red secondary metabolite prodigiosin, is reported with multiple color phenotypes (Zhou et al., 2016b). This bacterium is widely distributed in diverse environments, including water, soil, plants, and animals (Grimont and Grimont, 1978). In addition to the saprophytic life cycle, both mutualistic and parasitic roles of *S. marcescens* have been reported. *Serratia marcescens* may live in mutualistic symbiosis with fungi, plants, or animals. The mutualistic interactions between *S. marcescens* UENF-22GI and a biocontrol fungus *Trichoderma longibrachiatum* are shown not only beneficial for plant growth but also increased ecological fitness of the bacterium (de Andrade Reis et al., 2021). The *S. marcescens* strain NBRI1213 is beneficial to host plants by promoting growth and controlling a typical soil-borne plant pathogen *Phytophthora nicotianae* (Lavania et al., 2006). Several *S. marcescens* strains can colonize in the gut of mosquitoes and render host resistance to *Plasmodium* infection (Chen et al., 2017; Bai et al., 2019). *Serratia marcescens* is also a well-known opportunistic pathogen, which infects plants, invertebrates, and vertebrates. In plants, *S. marcescens* causes cucurbit yellow vine disease after invading into host phloem elements (Rascoe et al., 2003). Many reports indicate that the bacterium infects various invertebrates, mainly insects (Burritt et al., 2016; Zhou et al., 2016b), which causes deadly septicemia. *Serratia marcescens* has also been frequently reported as a common nosocomial infection pathogen (Schuppener et al., 2017). Like other bacterial pathogens, *S. marcescens* produces abundant virulence factors, such as extracellular enzymes including proteases, nucleases, lipases, lecithinase, hydrolytic enzymes, and toxins, etc. (Hines et al., 1988; Hejazi and Falkiner, 1997; Aucken and Pitt, 1998), which are controlled by transcriptional regulators, such as *rssAB*, *slyA*, and *eepR*, etc. (Ellison and Miller, 2006; Lin et al., 2010; Shanks et al., 2017). Previous reports have suggested that bacterial pigments are probably related to the pathogenic process (Liu and Nizet, 2009). However, unlike other pathogens, the red pigment prodigiosin seems not to be a necessary virulence factor that is required for *S. marcescens* pathogenesis (Zhou et al., 2016b), and its physiological or ecological roles remain largely unclear.

The prodigiosin produced by *S. marcescens* has a linear tripyrrole skeleton (2-methyl-3-pentyl-6-methoxyprodiginine), which is reported with antimicrobial, antialgal, antiprotozoal, antimalarial, antitumor, immunosuppressive, and antiviral activities (Williamson et al., 2007; Genes et al., 2011; Stankovic et al., 2014; Zhou et al., 2016c). The biosynthesis pathway of prodigiosin in *S. marcescens* has been well-studied (Williamson et al., 2006). Generally, the pathway can be divided into two parts which correlate with two major components, the 2-methyl-3-n-amyl-pyrrole (MAP) and 4-methoxy-2,2′-bipyrrole-5-carboxyaldehyde (MBC). In the presence of the enzyme PigC, MAP, and MBC are finally condensed to form the red pigment prodigiosin. The prodigiosin biosynthesis gene cluster (*Pig* gene cluster) consists of 14 opening reading frames (*pigA-N*), of which *pigB*, *pigD*, and *pigE* are responsible for the biosynthesis of MAP, while *pigA* and *pigF*-*N* are involved in the biosynthesis of MBC (Harris et al., 2004). Based on previous reports, all the *pig* genes are essential for prodigiosin synthesis. Williamson et al. (2005) have constructed several in-frame mutants of *pigA*-*N* in *Serratia*, and most of them (except for *pigK*) show reduced prodigiosin production. It has been proved that non-pigmented bacterial strains, such as *S. marcescens* Sma12 or other species, e.g., *Escherichia coli* XL-1, acquire prodigiosin-producing ability by gene transfer of the whole *Pig* gene cluster (Harris et al., 2004; Williamson et al., 2005; Coulthurst et al., 2006).

The biosynthesis of prodigiosin is modulated by various genetic and environmental factors. Several transcription factors have been revealed to involve in the prodigiosin biosynthesis process. The transcription regulators *pigP*, *slyA* (alternative: *rap*), *pigR*, *pigV*, *pigS*, *eepR*, and *hfq* show positive effects, while *CRP*, *pigX*, *pigU*, *hexS*, *pigT*, *rpoS*, *pigZ*, *copA*, *rsmA*, *rsmC*, *rcsB*, *metR*, and *cpxR* show negative effects (Williamson et al., 2006; Gristwood et al., 2008; Wilf et al., 2011; Wilf and Salmond, 2012; Hampton et al., 2016; Shanks et al., 2017; Pan et al., 2020a,b; Sun et al., 2020). Quorum sensing system, e.g., SpnIR and LuxS/AI-2, a global regulatory system, is also involved in prodigiosin biosynthesis in *S. marcescens*. SpnR usually acts as a negative regulator which is inhibited by AHLs that are synthesized by SpnI (Horng et al., 2002). LuxS/AI-2 often acts as a second quorum sensing system to control certain phenotypes (Sun et al., 2016). The two-component systems RssB/RssA, PigQ/W, and EnvZ/OmpR also regulate prodigiosin production in *Serratia* species (Fineran et al., 2005; Horng et al., 2010; Jia et al., 2021). It is worth noting that all these regulators may result in multiple unexpected phenotypic changes in *Serratia*, including biosurfactants, antibiotics and exoenzymes production, cell motility, and virulence. In addition, by using transposon insertion sequencing technology, several potential gene targets have been provided which might be involved in the prodigiosin synthesis of *S. marcescens* (Pan et al., 2019; Jia et al., 2021). Similar to other secondary metabolites, the biosynthesis of prodigiosin is extremely sensitive to environmental conditions. Many environmental factors, such as temperature, pH, oxygen concentration, media composition, ionic strength, light, and inorganic phosphate availability have been proved to affect prodigiosin production (Williamson et al., 2006). However, non-pigmented spontaneous mutants also arise, while the wild-type *S. marcescens* strains are subjected to continuous incubation under relatively stable laboratory conditions (Zhou et al., 2016b). It is interesting why *S. marcescens* loses pigment synthesis capacity in certain environments.

Both pigmented and non-pigmented *S. marcescens* strains naturally exist. According to the genetic data in the GenBank database, the majority of sequenced *S. marcescens* strains are non-pigmented which naturally lack the essential *Pig* gene cluster. Indeed, of all the 773 genome sequences of *S. marcescens* by July 7, 2021, there are only 51 strains contain the complete *Pig* gene cluster. Moreover, the largest proportion of non-pigmented isolates are obtained from clinical settings. We previously reported that the spontaneous non-pigmented mutants were derived from a wild-type *S. marcescens* strain during continuous cultivations *in vitro* (Zhou et al., 2016b). Except for the pigmentation features, the virulence of the mutants was very similar to each other. In the present study, we have found a growth difference among different color morphotypes. Transcriptomic and homologous-recombination-based gene knockout approaches were applied to explore why *S. marcescens* spontaneous non-pigmented strain has a growth advantage in laboratory conditions. According to our findings, *S. marcescens* both shuts down and activates several systems, including suppressing *pig* genes at the transcriptional level, to gain a growth advantage in certain conditions.

## Materials and Methods

### Microorganism, Culture, and Sub-Culture Conditions

In this study, the wild-type *S. marcescens* strain SCQ1 (CCTCC AB 2010221) was isolated from the hemolymph of diseased silkworm larva (*Bombyx mori*) previously, which was stored at −80°C (Zhou et al., 2016b). Unless mentioned specifically, all *S. marcescens* strains were routinely inoculated on Luria-Bertani (LB) agar (yeast extract 5 g/L, peptone 10 g/L, NaCl 10 g/L, and agar 20 g/L) or LB broth inoculated at 28°C. Where required, the medium was supplemented with ampicillin (50 μg/ml), chloramphenicol (50 μg/ml), and rifampicin (10 μg/ml).

The continuous sub-culture of the wild-type strain SCQ1 was performed. A single colony of SCQ1 was transferred to a 50 ml LB medium to prepare a seed culture with an OD_600_ of 0.6. The 1% of the seed culture was then transferred into a liquid LB medium (pH 7.0), and incubated at 28°C for 24 h with shaking (200 rpm) which was defined as the first passage of continuous sub-culture. Every 24 h, 500 μl of culture was transferred to 50 ml fresh LB broth. Before each sub-culture step, the samples were collected and 50 μl of diluted cultures were plated onto LB agar, incubated at 28°C for 48 h. The phenotypes of newly formed colonies were recorded and the proportions of color morphotypes were statistically analyzed. Triplicate experiments have been performed.

### Growth Curves and Prodigiosin Production Assays

The growth of all *S. marcescens* strains was measured in a Bioscreen C instrument (Growth Curves United States). The overnight cultures were adjusted to an OD_600_ of 0.6 using fresh LB medium, and 2 μl of which were inoculated in a 100-well honeycomb plate that each contained 198 μl of LB medium. In addition, 200 μl of the LB medium was added to three wells as blank controls. The honeycomb plate was placed in the Bioscreen C instrument (Growth Curves United States) incubated at 28°C and the OD_600_ was automatically measured every 2 h. The experiment was performed with three technical replicates and the growth curves were finally plotted with the growth time as the abscissa and the OD_600_ as the ordinate.

The assay of prodigiosin production was performed as follows: the colonies of *S. marcescens* were inoculated in 50 ml of LB medium and incubated at 28°C for 16 h with shaking (200 rpm). The 1 ml of each culture was harvested by centrifugation at 12,000 rpm for 5 min. The pellet was resuspended in 1 ml distilled water to measure the OD_600_ value and the supernatant was collected to measure the A_534_ value (Slater et al., 2003). The prodigiosin production was calculated and plotted as (A_534_/OD_600_). Triplicate experiments have been performed.

### Transcriptome Sequencing and Comparative Analysis of SCQ1 and SCQ1-3M

A single colony of SCQ1 and SCQ1-3M was transferred to an LB medium to prepare a seed culture with an OD_600_ value of 0.6. The 1% of seed culture was inoculated into 50 ml of LB medium and cultured at 28°C, 180 rpm for 8 h. Total RNA was extracted by the TRIzol-based method (Life Technologies, CA, United States). RNA degradation degree and potential contamination were monitored on 1% agarose gels. RNA purity and integrity were checked using NanoPhotometer® spectrophotometer (IMPLEN, CA, United States) and Bioanalyzer 2100 (Agilent, Santa Clara, CA, United States). The sequencing library was constructed using NEBNext® Poly (A) mRNA Magnetic Isolation Module (New England Biolabs, Ipswich, MA, United States). The clustering of the index-coded samples was performed on a cBot Cluster Generation System according to the manufacturer’s instructions. After cluster generation, sequencing was performed using the Illumina HiSeq™ 2500 platform with paired-end 150 base reads. The RNA-seq reads with adapters, with all A bases, with an N ratio > 10% and with low quality (number of bases with Q ≤ 20 was >50% among the entire reads) were removed to obtain the clean data. Then, the clean data were compared with ribosomal RNA in the *S. marcescens* genome to remove the corresponding ribosomal RNA, and the remaining reads were aligned to the SCQ1 reference genome using Bowtie2 (Version 2.2.8). Fragments per Kilobase of transcript per Million fragments (FPKM) mapped was used to present the quantification of gene expression and to eliminate the impacts of different gene lengths and sequencing depth. The edgeR package^1^ was used to identify differentially expressed genes (DEGs) across samples with fold changes (FC) ≥ 2 and a false discovery rate-adjusted *p* (*q*-value) < 0.05. DEGs were then subjected to enrichment analysis of GO function and KEGG pathways, and *q*-values were corrected using <0.05 as the threshold. Each sample was sequenced in three replications.

### Quantitative Real-Time RT-PCR

The total RNA was extracted using RNAiso Plus (Takara, China) according to the manufacturer’s protocol. The complementary DNA (cDNA) synthesis was carried out with RevertAid First Strand cDNA Synthesis Kit (Vazyme Biotechnology, Nanjing, China) according to the manufacturer’s instructions. Primer Premier 5.0 software was used to design primers, and *rpoB* was selected as the internal control gene (Supplementary Table S1). Reactions were performed using ChamQ SYBR qPCR Master Mix (Vazyme Biotechnology, Nanjing, China) in a 20 μl final volume containing 10 μl SYBR premix Ex Taq II, 0.8 μl of each primer, 0.4 μl ROX Reference Dye, 2 μl of diluted cDNA, and 6 μl sterile distilled water. Quantitative real-time RT-PCR (qRT-PCR) was performed on a StepOne™ Real-Time PCR System (48-well format; Applied Biosystem, United States) with the following program: 95°C for 10 min, followed by 40 cycles at 95°C for 15 s, at 60°C for 1 min. A melting curve analysis was performed to confirm product specificity. The relative expression levels were calculated using the 2^-ΔΔCT^ method. The FC in mRNA level were determined using the −ΔΔCt data analysis method. All samples were analyzed in three replications. The primers used in this study were listed in Supplementary Table S1.

### Strain Construction, Phenotypic and Virulence Investigation

The knockout plasmid of *slyA* was constructed by seamless cloning technology. The primers were designed using online software^2^ and were listed in Supplementary Table S1. Using *S. marcescens* SCQ1 genomic DNA as a template, the right-hand fragment and the left-hand fragment were amplified using primer pairs slyA-U-F/R and slyA-D-F/R, respectively. Amp-resistance fragment was amplified using Amp-F/R with pMD19-T as a template. The three fragments were ligated into *Sac*I/*Sal*I digested suicide plasmid pDM4 using ClonExpress MultiS One Step Cloning Kit (Vazyme Biotechnology, Nanjing, China) and then transformed into *E. coli* S17-1 to generate *E. coli* S17-1 (pDM4-*slyA*). The conjugative transfer was used to transform the knockout plasmid pDM4-*slyA* into *S. marcescens* SCQ1. Briefly, the *E. coli* S17-1 (pDM4-*slyA*) and SCQ1 were cultured in LB medium at 37°C and 28°C, respectively, to OD_600_ ≈ 0.6. The fermentation of *E. coli* S17-1 and SCQ1 were mixed at a ratio of 3:1 and the bacterial pellet was harvested by centrifugation at 12,000 rpm for 5 min. Then, the bacterial pellet was resuspended in 50 μl LB and 5 μl was transferred to LB plate containing a 0.22 μm membrane filter in the surface, which was cultured at 37°C for 4 h, followed by 28°C for 12–16 h. The colonies were spread on LB plates containing ampicillin, rifampicin, and chloramphenicol to obtain the single crossover recombinants SCQ1-pDM4-*slyA* which was verified by PCR using slyA-1F/R, slyA-2F/R. Overnight fermentation broth of SCQ1-pDM4-*slyA* was spread on LB plates containing 10% of sucrose and ampicillin without NaCl at 28°C for 24 h. The Δ*slyA* mutant was sucrose-resistant, ampicillin-resistant, and chloramphenicol intolerant and was further verified by PCR using slyA-1F/R, slyA-3F/R, and slyA-F/R and sequencing. To complement the mutants, the fragment containing the upstream non-coding region and the CDS of *slyA* was amplified using primer pairs slyA-hb-F/R and ligated into *XbaI*/*XhoI* digested vector pJQ200SK to generate complement plasmid pJQ200SK-*slyA*. Then, the plasmids pJQ200SK and pJQ200SK-*slyA* were transformed into *E. coli* S17-1 to producing *E. coli* S17-1-pJQ200SK and *E. coli* S17-1-pJQ200SK-*slyA*, respectively. As mentioned above, conjugative transfer was used to transform the pJQ200SK plasmid into SCQ1 and Δ*slyA* to generate SCQ1-pJQ200SK and Δ*slyA*-pJQ200SK, and the pJQ200SK-*slyA* plasmid was transformed into Δ*slyA* to producing Δ*slyA*-pJQ200SK-*slyA*. PCR (M13F/R) and sequencing were used for further verification.

The colony morphotypes, prodigiosin production, and growth of the Δ*slyA* mutant were investigated according to the methods mentioned above.

The healthy silkworm fifth-instar larvae reared with fresh mulberry leaves at 26°C were prepared for the experiments. Bacteria were harvested in the early exponential phase and counted by serial dilution methods which were expressed as colony-forming units (CFUs). The bacterial concentrations were adjusted to 5, 10, 25, and 50 CFU/μl with sterile insect saline (150 mM NaCl, 5 mM KCl, and 1 mM CaCl_2_), respectively. To assess the virulence of Δ*slyA* and SCQ1, 2 μl of each dilution was injected into silkworm hemocoel using a micro-injector, and sterile insect saline was used as control. Each group contained 30 larvae and the experiments were performed in triplicate. The dead larvae were continuously cultured at 26°C and the characteristics were recorded. The median lethal dose (LD_50_), median lethal time (LT_50_) values, and the statistical significance were evaluated using the SPSS software version 21.0 (SPSS, Inc./IBM, Armonk, NY, United States).

### Statistical Analyses

Data were presented as mean ± SD or mean ± SE as needed. Statistical analysis was performed using the SPSS software version 21.0 (SPSS, Inc./IBM, Armonk, NY, United States). When necessary, the student *t*-test was performed to compare the differences between the two groups. The *p*-value lower than 0.05 and 0.01 were designated as statistically significant (*) and highly significant (**), respectively.

## Results

### The Features of Different Morphotypes Derived From the *S. marcescens* SCQ1

**Figure 1:**
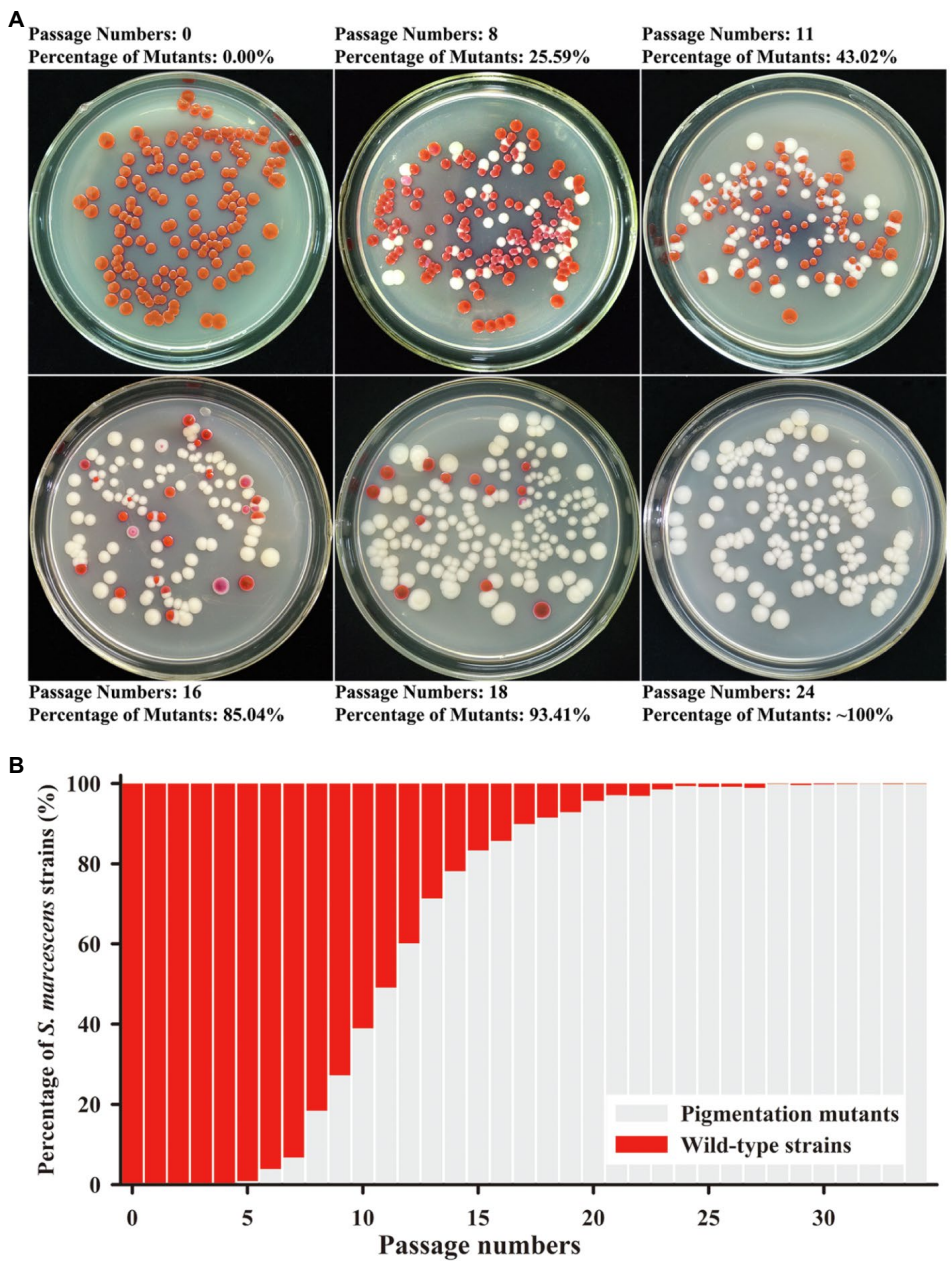
Mutation trend of *Serratia marcescens* during continuous passages. **(A)** Colonies on Luria-Bertani (LB) plate at the representative generations of passage. The percentage of color mutants is labeled upside. **(B)** The percentage of wild-type strains and mutants in each generation of passage.

The secondary metabolite prodigiosin endowed the *S. marcescens* wild-type strain SCQ1 with a red colony characteristic. Continuous cultivation of the wild-type strain led to different color morphotypes. As shown in Figure 1, the different color morphotypes were firstly detected in the broth at the 5th passage. From the 5–14th passage, the numbers of non-pigmented morphotypes increased dramatically which reached as high as 78% of the population. After the 24 passages, the population of the non-pigmented morphotypes exceeded 99% which indicated that the wild-type strains have been generally replaced (Figures 1A,B). During the passage processes, the spontaneous mutants with multiple color morphotypes were observed on the LB plate, including the colonies with a purple, pink, red center, or ring-shaped red color. However, most of the mutants displayed white colonies (Figure 1A).

### Growth and Prodigiosin Production of Different Color Morphotypes

**Figure 2:**
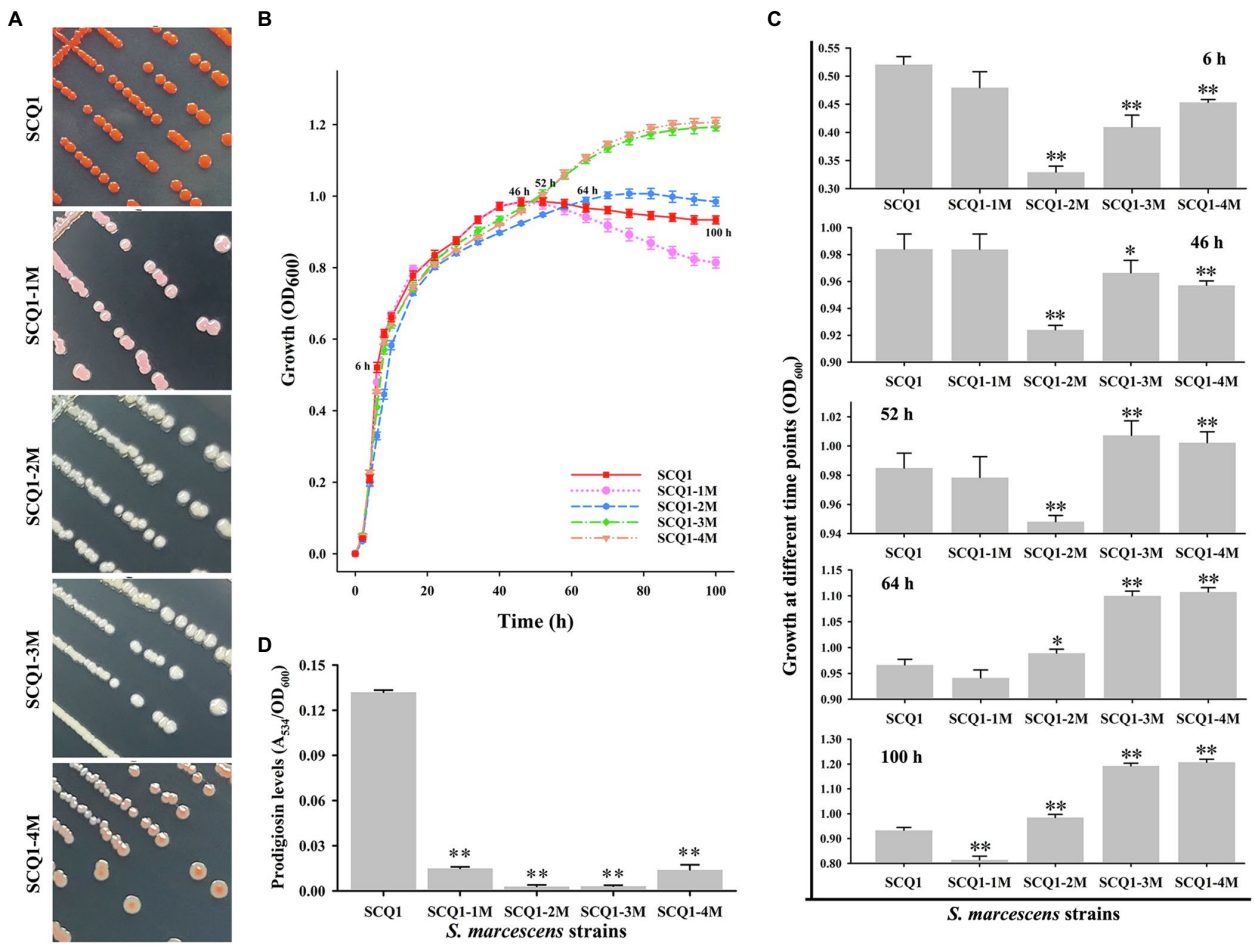
The phenotypic patterns of wild-type *S. marcescens* and its spontaneous mutants. **(A)** The colony of the wild-type strain SCQ1 and the four mutants on LB plate. **(B)** Growth of different *S. marcescens* strains in LB medium. The OD_600_ values are monitored every 2 h for 100 h using the Bioscreen C instrument with three technical replicates. Different lines and symbols represent different bacterial strains. The data are expressed as mean ± SD. Several time points are labeled and the details are shown in **(C)**. **(D)** The relative prodigiosin production in *different S. marcescens* strains is measured after 16 h incubation at 28°C. The values are calculated and expressed as mean ± SD (*n* = 3, ^*^p < 0.05, ^**^p < 0.01).

By further separation and purification, we have obtained four phenotypically stable mutants which were named SCQ1-1M (pink), SCQ1-2M (white), SCQ1-3M (white), and SCQ1-4M (red center; Figure 2A). The growth curves of the four morphotypes were distinctly different from the wild-type strain SCQ1 (Figure 2B). All tested strains entered the logarithmic growth phase after 2 h of inoculation. Within the first 50 h of culture, the growth trend of the pink strain was identical to the red wild-type strain, in which the OD_600_ of the SCQ1-1M reached the maximum value of 0.984 ± 0.011. The time point that the strain SCQ1 entered the stationary phase is about 52 h of cultivation, and the OD_600_ reached the highest value of 0.985 ± 0.01. SCQ1-1M entered the stationary phase a bit earlier than SCQ1 because the OD_600_ value was slightly decreased to 0.978 ± 0.014 at 52 h (Figure 2C). In contrast, the OD_600_ values of the other three color morphotypes were significantly lower than the strain SCQ1 at the period from 6 to 46 h (Figure 2C), but continuously increased and reached a higher value than that of the wild-type strain. Although, the cell densities of the three color morphotypes were eventually higher than the wild-type strain SCQ1, the time point was different which was delayed in the SCQ1-2M at 64 h (Figure 2C). Besides, the OD_600_ of the SCQ1-2M reached the maximum value of 1.007 ± 0.012 at 76 h, and then gradually decreased to 0.935 ± 0.017 at 100 h (Figure 2C). The OD_600_ of SCQ1-3M and SCQ1-4M were continuously increased within the cultivation period (Figure 2C). The prodigiosin productions of all the color morphotypes SCQ1-1M, SCQ1-2M, SCQ1-3M, and SCQ1-4M were significantly lower than the wild-type strain SCQ1, which decreased for 8.84, 45.76, 42.98, and 9.47-fold, respectively (Figure 2D). These results indicated that both bacterial growth and pigment production of the *S. marcescens* spontaneous mutants were changed, which suggested a complicated relationship between the two aspects.

### Expression Levels of *pigA-N* in the Four Mutants

**Figure 3:**
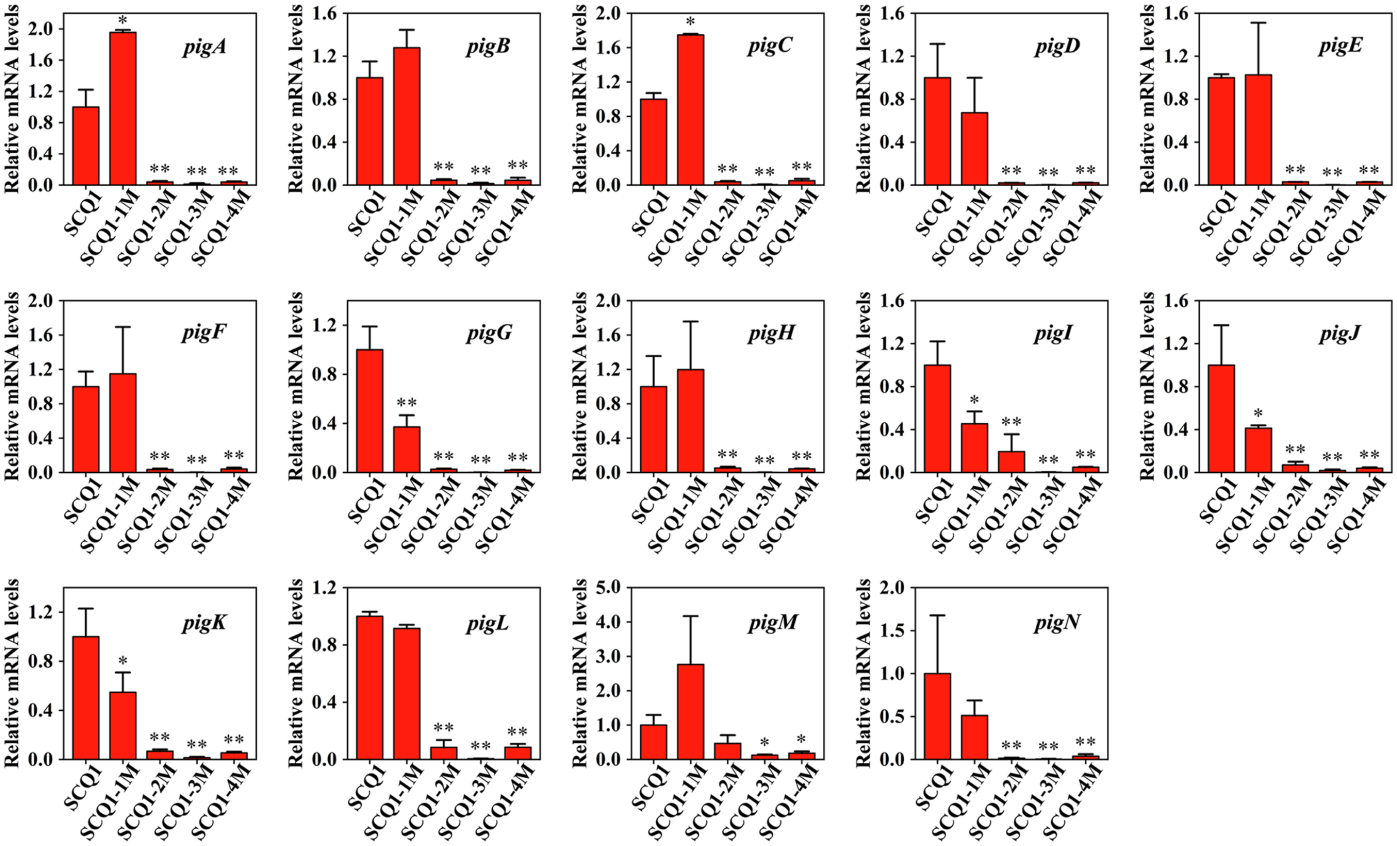
The mRNA expression levels of *pigA-pigN* in the wild-type strain SCQ1 and the four spontaneous mutants by quantitative real-time RT-PCR (qRT-PCR) analysis. Relative mRNA levels are quantified using *rpoB* as internal control and the data are displayed as mean ± SE (*n* = 3, ^*^p < 0.05, ^**^p < 0.01).

To determine the expression levels of the *pig* genes in the mutants, qRT-PCR analysis was performed (Figure 3). In SCQ1-1M, the expression levels of *pigA* and *pigC* were 1.954 ± 0.0313 and 1.747 ± 0.012, respectively. The values were even significantly higher than that of the wild-type strain SCQ1. However, the expression levels of *pigG*, *pigI*, *pigJ*, and *pigK* were significantly lower than that of in SCQ1, which were 0.372 ± 0.095, 0.454 ± 0.115, 0.413 ± 0.0258, and 0.548 ± 0.161, respectively. The expression levels of the other eight *pig* genes of SCQ1-1M were similar to SCQ1. In the SCQ1-2M, SCQ1-3M, and SCQ1-4M, the expression levels of *pigA-N* were all remarkably downregulated. The *pigD* is involved in the initial step of MAP biosynthesis, which the relative expression levels in the strains SCQ1-2M, SCQ1-3M, and SCQ1-4M were as low as 0.020 ± 0.0004, 0.001 ± 0.001, and 0.022 ± 0.001, respectively. The *pigI* is involved in the first step of MBC biosynthesis, which the relative expression levels in strains SCQ1-2M, SCQ1-3M, and SCQ1-4M were decreased to 0.195 ± 0.161, 0.003 ± 0.001, and 0.049 ± 0.004, respectively. The *pigC* is involved in the final step of prodigiosin biosynthesis, which the relative expression levels in strains SCQ1-2M, SCQ1-3M, and SCQ1-4M were 0.038 ± 0.009, 0.005 ± 0.004, and 0.051 ± 0.022, respectively. These results suggested that the *pig* genes of the spontaneous mutants were varied at the transcriptional level, and most of them were downregulated which was consistent with the phenotypic changes.

### Transcriptome Sequencing and Mapping Data

**Figure 4:**
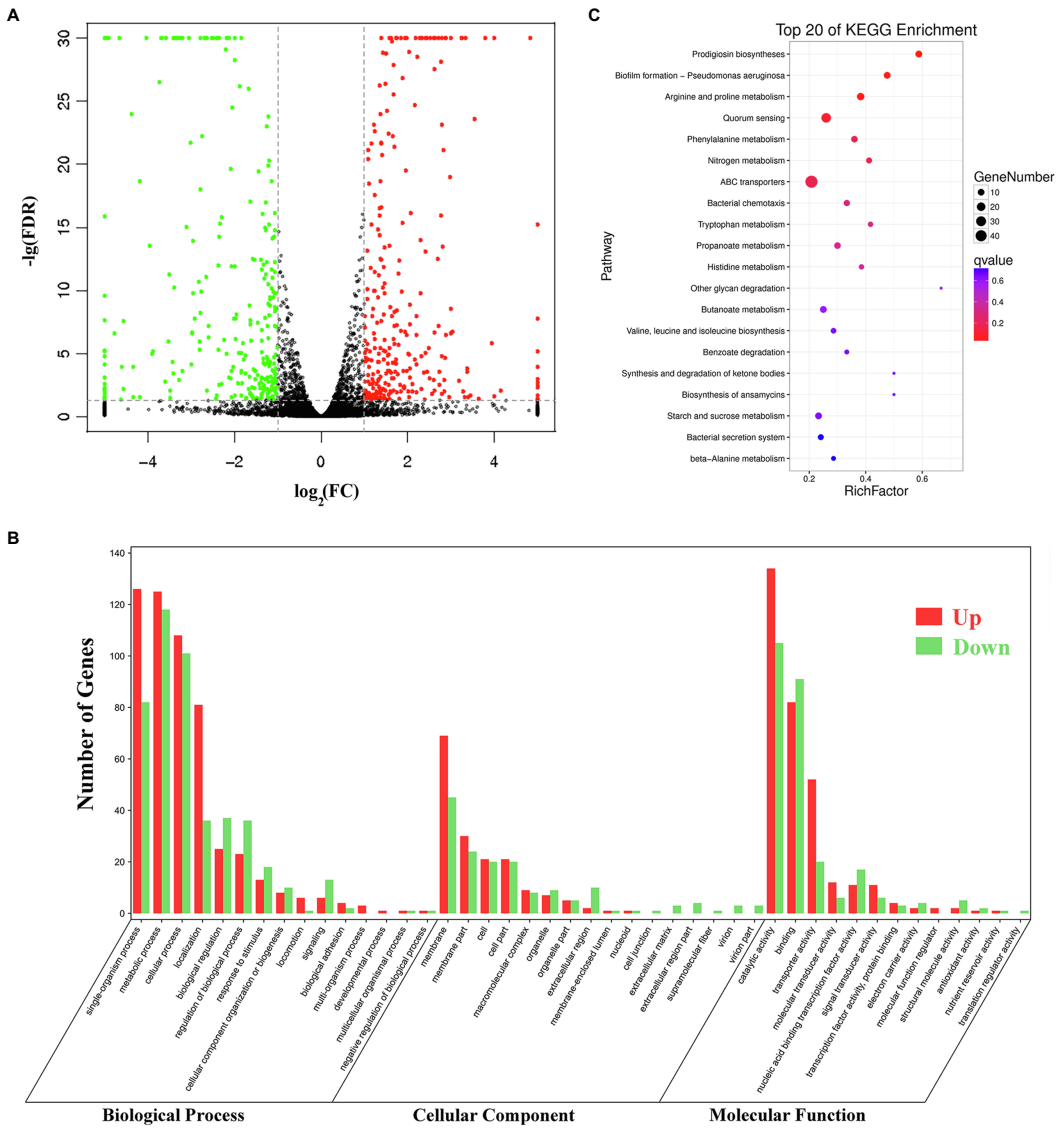
Transcriptomic analysis of the wild-type strain SCQ1 and the non-pigmented spontaneous mutant SCQ1-3M. **(A)** The volcano plot shows a general gene expression pattern of the SCQ1-3M. The red, green, and black dots represent significant upregulated, significant downregulated and, non-significant genes, respectively. The |log_2_(FC)| ≥ 1; FDR < 0.05 is set as the threshold for significantly DEGs. **(B)** Gene Ontology (GO) annotations of DEGs of the mutant SCQ1-3M. The DEGs are grouped into three main categories which are “cellular component,” “molecular function,” and “biological process” according to the GO data library. The red and green bars represent significant upregulated and downregulated DEGs, respectively. The Y-axis indicates the number of DEGs in each category. **(C)** The top 20 enriched KEGG pathways of DEGs. X- and Y-axes represent the Rich factor and the KEGG pathways, respectively.

*Pig* genes are regulated by various factors. Transcriptome sequencing was used to characterize the expression pattern of the whole genome, which may provide useful data for exploring the spontaneous pigmentation mutants. RNA-Seq data were generated from the pigmented wild-type strain SCQ1 and the non-pigmented mutant SCQ1-3M in biological triplicates. As the results, a total of 12,363,419,512 bp and 13,666,792,091 bp clean data were generated from Illumina HiSeq™ 2500 platform in SCQ1 and SCQ1-3M samples, respectively. The values of Q30 and the GC% were approximately 94 and 53% in each sample. After quality control and removing the rRNA, we obtained 68,491,508 and 77,474,952 clean reads in samples SCQ1 and SCQ1-3M (Table 1). The total reads of each sample were then mapped to the SCQ1 reference genome, and the ratio was higher than 96% (Table 1). After calculating and normalizing for FPKM, DEGs between SCQ1-3M and SCQ1 (SCQ1-3M vs. SCQ1) were identified. The volcano plot indicated the DEGs of SCQ1-3M vs. SCQ1 (Figure 4A). Of all the 4,692 genes, 582 DEGs with fold changes |log_2_(FC)| ≥ 1 and value of *q* < 0.05 were identified in SCQ1-3M when compared to SCQ1, in which the upregulated and downregulated DEGs were half and a half (Supplementary Table S2).

**Table 1:** Summary of transcriptome sequencing for SCQ1 and SCQ1-3M.

Sample	SCQ1	SCQ1-3M
Raw data (bp)	12,936,994,200	14,357,153,400
Clean data (bp)	12,363,419,512	13,666,792,091
GC%	53.30%	53.72%
Q30 (%)	94.69%	94.93%
Clean reads	86,061,358	95,559,656
Clean reads (ribosomal RNA were removed)	68,491,508	77,474,952
Mapped reads	66,690,253 (97.37%)	74,620,337 (96.32%)
Unique mapped reads	66,039,496 (96.42%)	73,740,130 (95.18%)
Multiple mapped reads	650,757 (0.95%)	880,207 (1.14%)

### qRT-PCR Validation

Quantitative real-time RT-PCR analysis was performed to validate the RNA-sequencing results. As shown in Supplementary Figure S1, 11 downregulated genes, including 4′-phosphopantetheinyl transferase (*pho*), O-methyltransferase (*pigF*), BsmB family protein BsmB (*BsmB*), 7-keto-8-aminopelargonate synthetase (*pigH*), Porin OmpC (*ompC*), ImpA family type VI secretion-associated protein (*vgrG1*), type VI secretion system (T6SS) effector, Hcp1 family (*hcp1*), type IV secretion protein Rhs (*vgrG*), transcriptional regulator slyA (*slyA*), transcriptional regulator (*tran*), KDP operon response regulator KdpE (*kdpE*), and 12 upregulated genes, including phenylacetate-CoA oxygenase subunit PaaB (*paaB*), succinylglutamate desuccinylase (*astE*), D-threitol dehydrogenase (*dthD*), periplasmic protein CpxP (*cpxP*), binding-dependent transport system inner membrane component family protein (*dppB*), phosphonate-transporting ATPase (*livF*), phenylacetic acid degradation protein (*paaE*), PTS system maltose and glucose-specific IICB components (*malX*), D-amino-acid oxidase (*mnmC*), ABC transporter substrate-binding protein (*dppA*), transcriptional regulator LrhA (*pecT*), and periplasmic substrate-binding component of an ABC superfamily oligopeptide transporter (*oppA*), were selected. In general, the expression characteristics of the tested genes were consistent with RNA-Seq transcriptomic analysis.

### Functional Annotation of DEGs

Gene Ontology (GO) assignments were used to classify the functions of the DEGs. A total of 3,328 reference genes assigned with GO terms were used as the background for enrichment analysis. The DEGs were assigned into 1,253 GO terms consisting of 698 biological process terms (353 DEGs), 413 molecular function terms (152 DEGs), and 142 cellular component terms (365 DEGs; Figure 4B). Most of the DEGs were annotated with more than one GO term. In the biological process group, the DEGs are enriched in “metabolic process” (68.84%), “cellular process” (59.21%), “single-organism process” (58.92%), and “organic substance metabolic process” (51.84%). In the molecular function group, the expression ratio of the DEGs in “catalytic activity” (65.48%) was the highest, followed by “binding” (47.4%) and “organic cyclic compound binding” (31.23%). In the cellular component group, the higher proportion of the DEGs involved in the “membrane” (75%) and “membrane part” (35.53%). KEGG assignments were performed to categorize gene functions. A total of 253 DEGs were mapped to 99 level-3 KEGG pathways, which were assigned into five KEGG level-1 groups, including “metabolism,” “cellular processes,” “environmental information processing,” “genetic information processing,” and “organismal systems.” The top 20 enriched KEGG pathways were shown in Figure 4C. Most of the DEGs were enriched in the “metabolic pathways” (39.53%), “ABC transporters” (19.37%), “microbial metabolism in diverse environments” (16.21%), “biosynthesis of antibiotics” (13.83%), “biosynthesis of secondary metabolites” (13.44%), and “quorum sensing” (10.28%). Based on the *q*-value, three pathways were significantly enriched, including “prodigiosin biosynthesis,” “biofilm formation,” and “arginine and proline metabolism” (Supplementary Table S3). These results suggested that the DEGs involved in metabolism, membrane proteins, biosynthesis of secondary metabolites, and transporters might play important roles in spontaneous non-pigmented mutants.

### Transcription Patterns of Several Pathways in Spontaneous Non-pigmented Mutant

#### Transcription Factors

Numerous studies have shown that the biosynthesis of prodigiosin was regulated by a variety of transcription factors. In this study, a total of 22 transcriptional factors were detected (Table 2). There were five transcription regulators, including *hexS*, *cdaR*, *lrp*, *malT*, and *feaR*, which were upregulated with the fold change values a biter higher than 1.0. In contrast, the remaining 17 regulators were significantly downregulated (Table 2). SMR_GM001072 and SMR_GM004456 showed the highest fold changes that were downregulated as −13.34 and −12.70, respectively. Besides, the fold changes of the most downregulated genes were above −1.5, which included two previously reported prodigiosin-synthesis-related regulators, *slyA* and *pigP*, and the values were −2.09 and −1.66, respectively. These transcriptional regulators might possess direct or indirect effects related to the prodigiosin biosynthesis pathway in the spontaneous mutant SCQ1-3M.

**Table 2:** Transcriptional regulators in differentially expressed genes (DEGs).

Gene ID	Descriptions	log_2_(FC)
SMR_GM004504	Transcriptional regulator HexS	1.39
SMR_GM002200	XRE family transcriptional regulator CdaR	1.25
SMR_GM002802	AsnC family transcriptional regulator Lrp	1.16
SMR_GM000986	Transcriptional regulator MalT	1.13
SMR_GM002061	Transcriptional regulator FeaR	1.10
SMR_GM001072	Transcriptional regulator	−13.34
SMR_GM004456	TetR family transcriptional regulator	−12.70
SMR_GM003448	Rrf2 family transcriptional regulator	−4.18
SMR_GM004630	LysR family transcriptional regulator AmpR	−3.89
SMR_GM000608	GntR family transcriptional regulator	−2.80
SMR_GM003301	Transcriptional regulator SlyA	−2.09
SMR_GM002086	Transcriptional regulator	−1.90
SMR_GM001130	Transcriptional regulator	−1.88
SMR_GM002619	LuxR family transcriptional regulator	−1.83
SMR_GM004490	LysR family transcriptional regulator YafC	−1.80
SMR_GM000582	Transcriptional regulator PigP	−1.66
SMR_GM002625	AraC family transcriptional regulator Rob	−1.62
SMR_GM001009	RpiR family transcriptional regulator	−1.62
SMR_GM004297	LacI family transcriptional regulator YvdE	−1.53
SMR_GM000367	DeoR family transcriptional regulator SrlR	−1.40
SMR_GM004007	AraC family transcriptional regulator	−1.13
SMR_GM004594	DNA-binding transcriptional regulator DsdC	−1.04

#### Membrane Proteins

It has been investigated that the membrane vesicles which consisted of essential membrane proteins were prodigiosin storage and secretion device. In transcriptome sequencing, a total of 26 membrane proteins were identified (Table 3). There are five DEGs, including *ompF* (SMR_GM002838, SMR_GM002556, and SMR_GM003927), *ompW* (SMR_GM003755), *membrane protein* (SMR_GM002333), and *ydcZ* (SMR_GM004201) were upregulated with fold changes ranging from 1.1 to 2.61. The other 21 membrane proteins were downregulated in SCQ1-3M with fold changes ranging from −1.02 to −4.56. The membrane proteins SMR_GM002390 and SMR_GM001605 were downregulated with maximum fold changes of −13.20 and −13.02, respectively. The changes of membrane proteins might be related to the storage and transportation of bacterial prodigiosin.

**Table 3:** Membrane proteins in DEGs.

Gene ID	Descriptions	log_2_(FC)
SMR_GM002390	Membrane protein	−13.20
SMR_GM001605	Membrane protein	−13.02
SMR_GM004286	Membrane protein YghQ	−4.56
SMR_GM000583	Membrane protein	−3.35
SMR_GM004458	Porin OmpC	−3.31
SMR_GM000228	Membrane protein YphA	−3.06
SMR_GM003299	Membrane protein AaeA	−2.36
SMR_GM000059	Membrane protein YeaQ	−2.17
SMR_GM002325	Membrane protein YbfA	−2.09
SMR_GM002592	Outer membrane protein X OmpX	−1.99
SMR_GM001806	Membrane protein Smp	−1.55
SMR_GM004141	Membrane protein	−1.45
SMR_GM003137	Inner membrane protein	−1.43
SMR_GM000034	Membrane protein Spro_3503	−1.19
SMR_GM001890	Membrane bound cell division protein FtsL	−1.18
SMR_GM001076	Inner membrane protein YhjD	−1.15
SMR_GM004140	Membrane protein YeaQ	−1.09
SMR_GM000239	Membrane protein ElaB	−1.07
SMR_GM001352	Membrane protein BcsG	−1.07
SMR_GM003158	Membrane protein	−1.02
SMR_GM002838	Outer membrane pore protein N OmpF	1.10
SMR_GM003755	Outer membrane protein W OmpW	1.22
SMR_GM002556	Porin OmpF	1.43
SMR_GM002333	Membrane protein	1.45
SMR_GM004201	Membrane protein YdcZ	1.55
SMR_GM003927	Outer membrane protein C OmpF	2.61

#### Type VI Secretion System

Type VI secretion system is a weapon used by Gram-negative bacteria to fight against other bacterial competitors. As in the wild-type strain SCQ1, *S. marcescens* possessed a T6SS that contains 13 core proteins. In the transcriptome sequencing results, all the DEGs related to T6SS, including 11 core components, were downregulated in SCQ1-3M with fold changes ranging from −1.02 to −5.16 (Table 4). The membrane-anchoring complex subunit TssM was downregulated in SCQ1-3M with a fold change of −4.57. DotU, TssK, and TssF which were the main components of the T6SS baseplate complex showed the fold changes of −4.19, −2.80, and −2.65, respectively. The *tssA* which encoded a dodecamer complex had a fold change value of −2.97. The results suggested a metabolic tradeoff between a high-cost system and growth in *S. marcescens*. Under artificial conditions which lacked competitors, cutting down T6SS would save resources that benefit bacterial growth.

**Table 4:** Differentially expressed genes involved in type VI secretion system (T6SS).

Gene ID	Descriptions	log_2_(FC)
SMR_GM004066	Type VI secretion system tip protein VgrG	−3.74
SMR_GM004075	Type VI secretion system baseplate subunit TssK	−2.80
SMR_GM004076	DotU family type VI secretion system protein	−4.19
SMR_GM004077	Type VI secretion system membrane subunit TssM	−4.57
SMR_GM004078	Type VI secretion system-associated protein	−4.61
SMR_GM004079	Type VI secretion system protein TssA	−2.97
SMR_GM004080	Type VI secretion system contractile sheath small subunit ImpB	−2.75
SMR_GM004081	Type VI secretion system contractile sheath large subunit ImpC	−2.78
SMR_GM004082	Type VI secretion system tube protein Hcp	−3.41
SMR_GM004083	Type VI secretion protein	−3.20
SMR_GM004084	Type VI secretion system-associated FHA domain protein TagH	−4.38
SMR_GM004085	Type VI secretion system, serine/threonine protein phosphatase	−4.78
SMR_GM004086	Type VI secretion system-associated protein	−5.16
SMR_GM004087	Protein of avirulence locus ImpE	−13.5
SMR_GM004089	Type VI secretion system baseplate subunit TssF	−2.65
SMR_GM004091	Type VI secretion system ATPase TssH	−3.44
SMR_GM004093	Type VI secretion system tip protein VgrG	−2.96
SMR_GM004095	Type IV secretion protein Rhs	−1.32

#### Amino Acid Metabolism and Transport

In the DEGs of SCQ1-3M vs. SCQ1, we have found several enzymes which could be classified into 11 amino acid metabolic pathways, including “Arginine and proline metabolism (ko00330),” “Phenylalanine metabolism (ko00360),” “Tryptophan metabolism (ko00380),” “Histidine metabolism (ko00340),” “beta-Alanine metabolism (ko00410),” “Alanine, aspartate and glutamate metabolism (ko00250),” “Valine, leucine and isoleucine degradation (ko00280),” “Tyrosine metabolism (ko00350),” “Lysine degradation (ko00310),” “Glycine, serine and threonine metabolism (ko00260),” and “Cysteine and methionine metabolism (ko00270)” (Table 5; Supplementary Table S4). Most DEGs in these pathways were upregulated. The enhanced amino acid degradation ability reminded us to analyze the closely related pathways, e.g., amino acid import pathways. Several DEGs involved in amino acid and peptide transport pathways were generally upregulated, including “oligopeptide transport (Opp) system,” “dipeptide transport (Dpp) system,” “histidine transport system,” “arginine transport system,” and “branched chain amino acid transport system” (Table 6). These results suggested that the spontaneous mutant SCQ1-3M imported extra amino acids for further degradation.

**Table 5:** Summary of DEGs involved in amino acid metabolism.

Pathway	Number of DEGs
Phenylalanine metabolism	9
Arginine and proline metabolism	13
Tryptophan metabolism	5
Histidine metabolism	5
Beta-Alanine metabolism	4
Alanine, aspartate, and glutamate metabolism	7
Valine, leucine, and isoleucine degradation	4
Tyrosine metabolism	5
Lysine degradation	3
Glycine, serine, and threonine metabolism	7
Cysteine and methionine metabolism	6

**Table 6:** Differentially expressed genes involved in amino acid and peptide transport system.

Gene ID	Descriptions	log_2_(FC)
SMR_GM003688	oppA, mppA; oligopeptide transport system substrate-binding protein	1.73
SMR_GM003779	oppF; oligopeptide transport system ATP-binding protein	1.96
SMR_GM003781	oppC; oligopeptide transport system permease protein	1.58
SMR_GM003782	oppB; oligopeptide transport system permease protein	1.00
SMR_GM003783	oppA, mppA; oligopeptide transport system substrate-binding protein	1.95
SMR_GM003784	oppA, mppA; oligopeptide transport system substrate-binding protein	1.38
SMR_GM004627	dppA; dipeptide transport system substrate-binding protein	1.81
SMR_GM001347	dppA; dipeptide transport system substrate-binding protein	2.32
SMR_GM001348	dppB; dipeptide transport system permease protein	2.71
SMR_GM001349	dppC; dipeptide transport system permease protein	1.92
SMR_GM001350	dppD; dipeptide transport system ATP-binding protein	2.20
SMR_GM001351	dppF; dipeptide transport system ATP-binding protein	2.77
SMR_GM004526	hisP; histidine transport system ATP-binding protein	1.28
SMR_GM004528	hisQ; histidine transport system permease protein	1.98
SMR_GM004529	hisJ; histidine transport system substrate-binding protein	1.88
SMR_GM002770	artJ; arginine transport system substrate-binding protein	1.24
SMR_GM002773	artI; arginine transport system substrate-binding protein	1.35
SMR_GM001434	livF; branched-chain amino acid transport system ATP-binding protein	−1.23
SMR_GM004266	livF; branched-chain amino acid transport system ATP-binding protein	2.85
SMR_GM004267	livG; branched-chain amino acid transport system ATP-binding protein	1.41
SMR_GM004268	livM; branched-chain amino acid transport system permease protein	2.01
SMR_GM004269	livH; branched-chain amino acid transport system permease protein	2.50
SMR_GM004270	livK; branched-chain amino acid transport system substrate-binding protein	2.42

### *slyA* Affected the Prodigiosin Biosynthesis, Bacterial Virulence, and Growth

**Figure 5:**
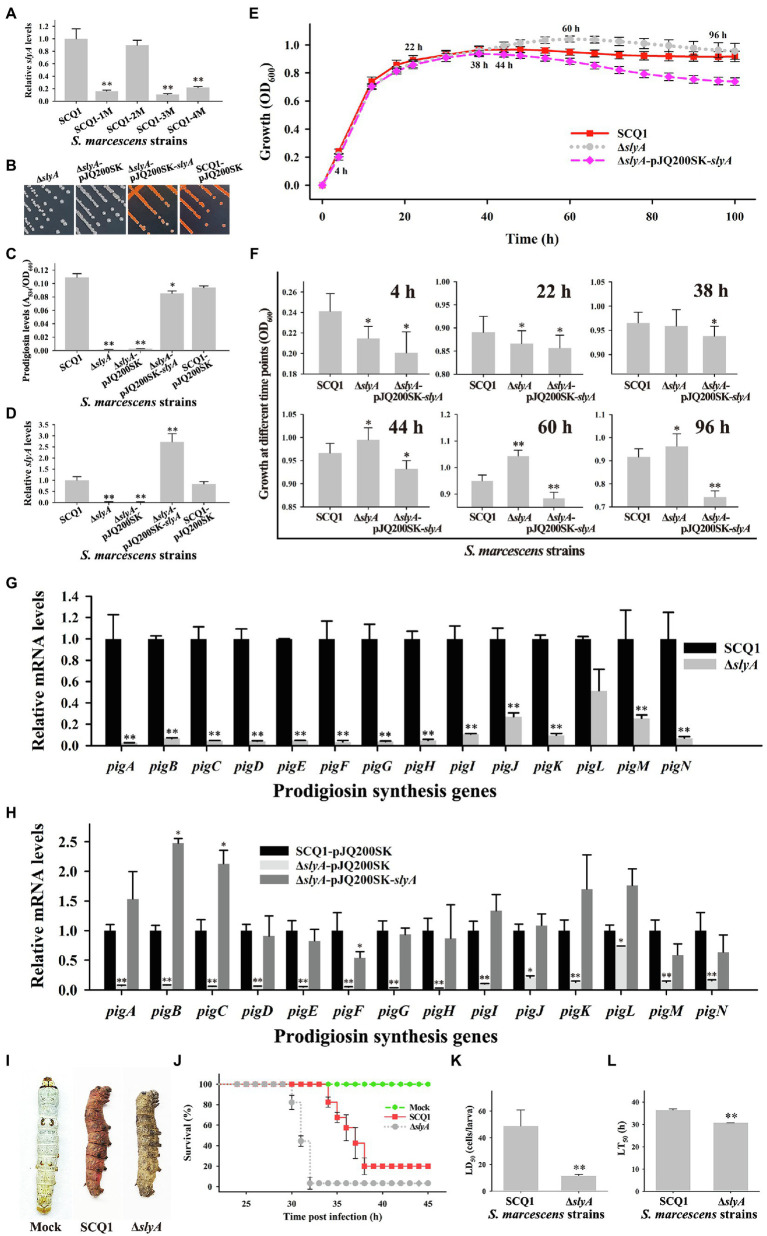
The characteristics of the Δ*slyA* mutant. **(A)** The mRNA expression level of *slyA* in different spontaneous mutants. **(B)** Colonies of the mutant Δ*slyA*, the control strains Δ*slyA*-pJQ200SK and SCQ1-pJQ200SK, and the complemented strain Δ*slyA*-pJQ200SK-*slyA* on LB plate inoculated at 28°C for 36 h. **(C)** The relative prodigiosin production levels in the strain SCQ1, Δ*slyA*, Δ*slyA*-pJQ200SK, Δ*slyA*-pJQ200SK-*slyA*, and SCQ1-pJQ200SK are measured after 16 h incubation at 28°C. The A_534_/OD_600_ values are calculated and expressed as mean ± SD. **(D)** The mRNA expression level of *slyA* in the recombinant strains. **(E)** The growth curves of the strain SCQ1, Δ*slyA*, and Δ*slyA*-pJQ200SK-*slyA*. The OD_600_ values are monitored every 2 h for 100 h using the Bioscreen C instrument with three technical replicates. Different lines and symbols represent different bacterial strains. The data are expressed as mean ± SD. Several time points are labeled and the details are shown in **(F)**. **(G)** The mRNA expression levels of *pigA-pigN* in the wild-type strain SCQ1 and the mutant Δ*slyA*. **(H)** The mRNA expression levels of *pigA-pigN* in Δ*slyA*-pJQ200SK-*slyA* and the control strains with an empty plasmid pJQ200SK. **(I)** Symptom of silkworm larvae infected by the strain SCQ1 and Δ*slyA*. The mock group represents silkworm larvae injected with sterile saline as the negative control. **(J)** Cumulative survival of silkworm larvae injected with SCQ1 [100 colony-forming units (CFUs) per larva], Δ*slyA* (100 CFUs per larva), and sterile saline. **(K)** The LD_50_ values of the strain SCQ1 and Δ*slyA* in the silkworm larvae. **(L)** The LT_50_ of the strain SCQ1 and Δ*slyA* in the silkworm larvae. The data were expressed as mean ± SD (*n* = 3, ^*^p < 0.05, ^**^p < 0.01).

The growth advantage of color mutants is related to several genes. The transcriptional regulator *slyA* was significantly downregulated in the non-pigmented mutant SCQ1-3M. However, different strains had distinct expression patterns of *slyA* (Figure 5A), which suggested changes in the regulation of prodigiosin biosynthesis. Nonetheless, *slyA* was reported as a relative downstream positive regulator of the *pig* gene cluster. Therefore, we have constructed a *slyA* knockout mutant to investigate whether downregulated *pig* genes directly led to a similar growth advantage. The Δ*slyA* mutant showed a white colony, increased virulence and growth rate. Just like the SCQ1-3M, the Δ*slyA* mutant could not synthesize prodigiosin which was distinct from the wild-type strain SCQ1 (Figure 5B). The relative prodigiosin production of the Δ*slyA* mutant was 0.0015 ± 0.0002 after 16 h cultivation, which was decreased by about 70-fold compared with the SCQ1 (Figure 5C). The Δ*slyA* mutant was complemented by the wild-type *slyA* gene on the recombinant plasmid pJQ200SK-*slyA*. An empty plasmid pJQ200SK was transformed into both SCQ1 and Δ*slyA* as controls (Figure 5D). The prodigiosin production in the complemented mutant was largely restored (Figure 5C). The results indicated that *slyA* was responsible for prodigiosin dyssynthesis in SCQ1-3M but might not be responsible for that in other spontaneous mutants.

The growth pattern of the Δ*slyA* mutant was also varied. During 4–22 h of cultivation, the OD_600_ of the Δ*slyA* mutant was significantly lower than that of the SCQ1, but the value increased quickly and exceeded that of the SCQ1 in the further 44–100 h with a maximum of 1.043 ± 0.022 at 60 h (Figures 5E,F). The growth pattern of the control strain SCQ1-pJQ200SK and Δ*slyA*-pJQ200SK was similar to the SCQ1 and Δ*slyA* (data not shown), respectively. The complemented mutant Δ*slyA*-pJQ200SK-*slyA* showed a lower growth rate compared with the wild-type strain (Figures 5E,F). The maximum value of OD_600_ in Δ*slyA*-pJQ200SK-*slyA* was 0.938 ± 0.021 at 38 h (Figure 5F), and it was gradually decreased. The reason was unclear, but possibly due to the varied expression level of the transcriptional regulator *slyA*. qRT-PCR analysis indicated that the decreased prodigiosin yield was caused by suppressed pig genes (Figure 5G). However, in Δ*slyA*-pJQ200SK-*slyA*, the expression level of *slyA* was higher than the wild-type strain SCQ1 and the negative control SCQ1-pJQ200SK, which might cause unexpected effects. As shown in Figure 5H, Δ*slyA*-pJQ200SK-*slyA* showed a relatively high expression level of *pig* genes, in which the *pigB* and *pigC* were even higher than the negative control SCQ1-pJQ200SK. Nonetheless, Δ*slyA* mutant had a higher cell density than the wild-type strain SCQ1 for a long period, which indicated a better growth at laboratory conditions.

Then, we evaluated the virulence of the Δ*slyA* mutant and found it was increased compared with the wild-type strain SCQ1. Both the Δ*slyA* and SCQ1 caused the rapid death of silkworms (*Bombyx mori*), but with a different symptom in which SCQ1-infected larvae showed red color, while Δ*slyA*-infected ones exhibited dark brown color (Figure 5I). Briefly, most of the inoculated larvae were dead within 38 h, and the lethal time of the Δ*slyA* mutant was shorter than that of the SCQ1 (Figure 5J). The LD_50_ value of the Δ*slyA* mutant was 9.977 ± 3.105 (cells/larva) which was decreased by 4.89-fold compared with 48.83 ± 11.56 (cells/larva) of SCQ1 (Figure 5K). The LT_50_ value of Δ*slyA* (30.68 h) was also lower than SCQ1 (36.34 h; Figure 5L). Despite the higher virulence suggesting a higher cost of resources, Δ*slyA* mutant still possessed a growth advantage compared with the wild-type strain under laboratory conditions.

## Discussion

The bacterial prodigiosin is synthesized by the biosynthetic gene cluster that contains 14 *pig* genes (Harris et al., 2004). In *S. marcescens*, both pigmented and non-pigmented strains are widely distributed in the environment. What happens to the *pig* genes in non-pigmented or less pigmented strains? Based on the sequence data from the GeneBank database, several non-pigmented strains are lacking one or more *pig* genes, even the entire gene cluster.^3^ In laboratory conditions, we have observed that spontaneous non-pigmented mutants of *S. marcescens* frequently occurred during the continuous propagation or prolonged incubation (Figure 1). This phenomenon of the *S. marcescens* SCQ1 is similar to the previous reports of *S. marcescens* 274 and HY (Bunting, 1940; Labrum and Bunting, 1953). It seems very easy for *S. marcescens* strains to lose prodigiosin synthesis capacity. However, the spontaneous reversion was not observed under the conditions tested, which was quite different compared with the previous studies (Bunting, 1946). Previously, we have sequenced the four color morphotypes and the parent strain SCQ1 (Xiang et al., 2021). Surprisingly, the *Pig* gene cluster (both sequences and positions) is completely preserved among all sequenced strains, and neither insertions nor deletions are detected. The results reminded us that the changes might occur at the transcriptional level. The qRT-PCR results strongly supported this hypothesis that the expression levels of *pig* genes in the mutants SCQ1-2M, SCQ1-3M, and SCQ1-4M were significantly downregulated (Figure 3). The SCQ1-1M was an exception because it expressed some *pig* genes at a relatively high level, e.g., the expression levels of *pigA* and *pigC* are even higher than that of the parent strain (Figure 3). The highly expressed genes suggested a higher cost of resources. However, downregulation or loss-of-function variant in a single *pig* gene can cause prodigiosin dyssynthesis (Williamson et al., 2006). In SCQ1-1M, four out of 14 *pig* genes were significantly downregulated which would decrease prodigiosin production. Thus, under laboratory conditions, the reduced pigment production of *S. marcescens* can be directly ascribed to the suppressed expression of the *pig* genes.

The different spontaneous color morphotypes of *S. marcescens* bring the question of whether they have any physiological differences. During the sub-culturing processes, we found that the non-pigmented morphotypes rapidly replaced nearly all the wild-type cells within 24 passages (Figure 1B). The results indicated that the non-pigmented morphotypes possessed a competitive growth advantage over the parent strain. The growth curves showed a higher cell density of the mutants SCQ1-2M, SCQ1-3M, and SCQ1-4M at the stationary phase compared with the red parent strain. The result was correlated with the lower expression level of *pig* genes as mentioned above. The biosynthesis of the red pigment prodigiosin is controlled by transcriptional regulators. In the present study, 22 differentially expressed regulators were identified in the strain SCQ1-3M (Table 2). However, only three of them, including the repressor *hexS*, the positive regulators *pigP* and *slyA*, were proved involved in prodigiosin synthesis (Williamson et al., 2006; Shanks et al., 2013). According to previous reports, the *hexS* regulated *pigP* (Shanks et al., 2013), and the latter regulated *slyA* (Williamson et al., 2006). The *slyA* is a relative downstream regulator in prodigiosin biosynthesis; however, it shows different expression patterns among spontaneous mutants (Figure 5A). The results suggested different color mutants might have different regulatory features. Nonetheless, in SCQ1-3M, it is possible that downregulated *slyA* directly led to prodigiosin dyssynthesis, and might bring growth advantage. To test the hypothesis, we have constructed a *slyA* deletion mutant. The Δ*slyA* mutant showed greatly reduced prodigiosin production as previously reported (Fineran et al., 2005; Pan et al., 2020a). Within a long period from 44 to 96 h, the cell density of the Δ*slyA* mutant was significantly higher than that of the wild-type strain SCQ1, which indicated a growth advantage. However, transcription regulators usually have multiple roles, e.g., *slyA* is involved in many physiological activities (Ellison and Miller, 2006). In this study, the growth advantage was not observed in the complemented strain Δ*slyA*-pJQ200SK-*slyA* (Figures 5E,F). Although the details were not clear, it might be caused by differentially expressed *slyA* (Figure 5D), which also led to the varied expression level of *pig* genes (Figure 5H). The phenomenon that highly expressed or overexpressed *pig* genes correlated with quickly decreased cell density was quite similar to that in the SCQ1-1M (Figures 2B, 3). Because *slyA* is also involved in the virulence in many bacterial species (Libby et al., 1994; Haque et al., 2009; Michaux et al., 2011; Zhou et al., 2016a), we also used silkworm larvae as the animal model to assess the virulence of the Δ*slyA* mutant. Unexpectedly, the virulence of Δ*slyA* mutant was increased, which suggested a higher cost of resources. Despite that, the Δ*slyA* mutant showed better growth than the wild-type strain as mentioned above. In general, the downregulated *pig* genes that resulted in non-pigmented mutants would bring a competitive growth advantage.

**Figure 6:**
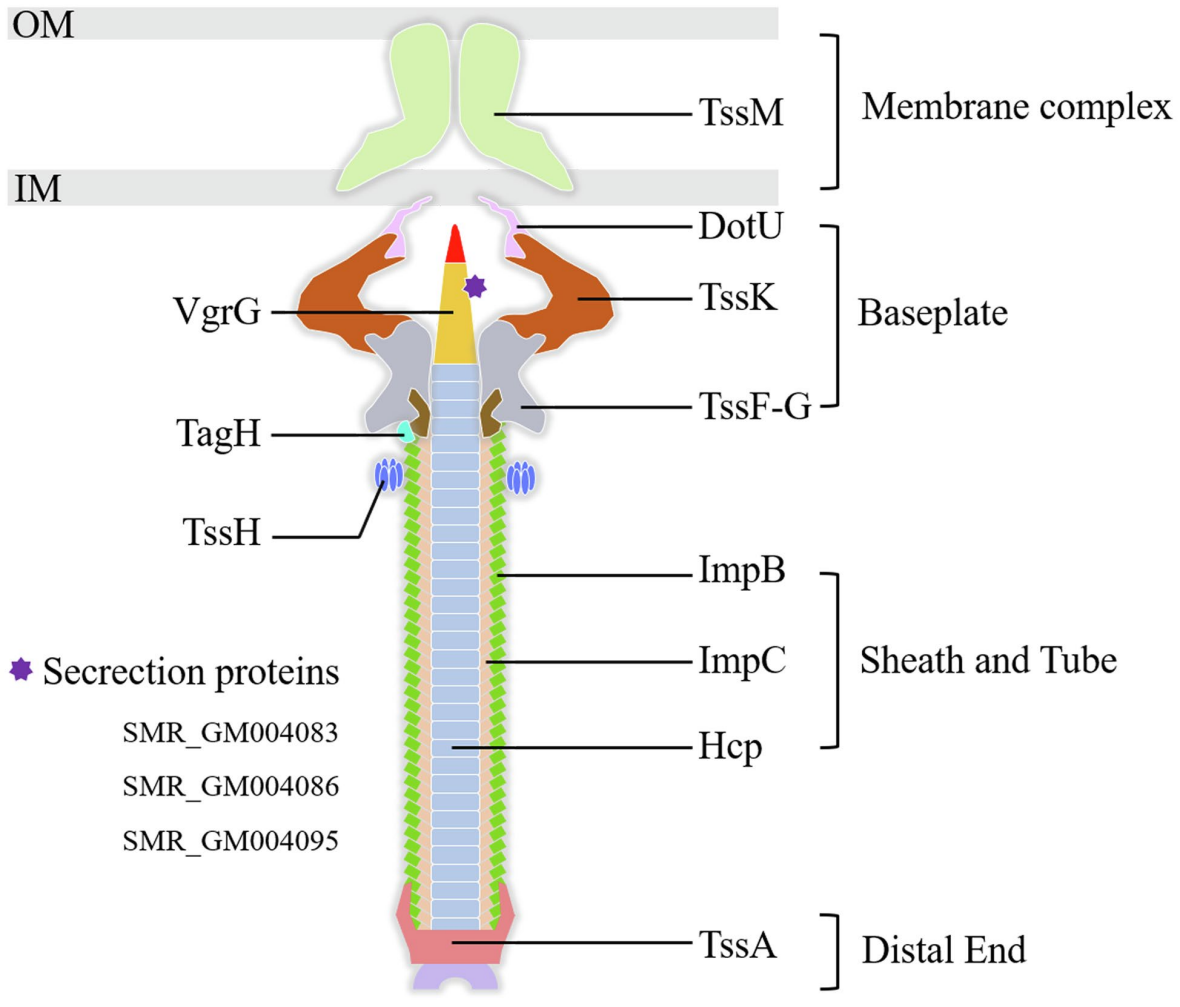
The components of the T6SS detected in the present study. All the labeled genes are significantly downregulated in the spontaneous non-pigmented mutant SCQ1-3M. The DEGs of T6SS related secretion proteins in the present study were listed at lower-left. OM, outer membrane; IM, inner membrane.

Pigment dyssynthesis was not the only factor that contributed to bacterial growth because the growth differences existed among the tested strains. The transcriptomic analysis also indicated that membrane proteins, T6SS, amino acid metabolism, and amino acid and peptide transport pathways were significantly varied. The membrane proteins are high-cost components that are involved in prodigiosin synthesis, storage, and exocytosis processes (Williamson et al., 2006; Chawrai et al., 2012; McMahon et al., 2012). McMahon et al. (2012) have found at least 13 outer membrane vesicle (OMV) proteins in *S. marcescens*. In the present study, downregulated membrane proteins, e.g., OmpX and OmpC, would hinder OMV formation which might cause feedback inhibition of prodigiosin synthesis (Pan et al., 2019). The T6SS is a high-cost nanomachine used by Gram-negative bacteria with multiple functions (Silva et al., 2020). In this study, we have found 18 downregulated T6SS-related genes in the spontaneous mutant SCQ1-3M, including most of the core components (Figure 6). Numerous studies indicate that T6SS generally implicates virulence in pathogenic bacteria (Miyata et al., 2011; Repizo et al., 2015; Hu et al., 2019). However, it has been proved that T6SS is not a virulence factor in *S. marcescens* (Murdoch et al., 2011). Previously, we have reported that the spontaneous non-pigmented mutants showed similar pathogenicity to silkworm larvae as the wild-type strain *S. marcescens* (Zhou et al., 2016b). In addition, a mutation in T6SS was suggested involving *S. marcescens* pigment production by using Tn5-mutagenesis technology (Jia et al., 2021). The downregulated T6SS might relate to the reduced prodigiosin production, or at least did not affect bacterial virulence. In addition to the suppressed high-cost systems, both amino acid degradation and import pathways were activated. The biosynthesis of prodigiosin relies on several amino acids, including proline, serine, alanine, and methionine (Williamson et al., 2006). Because the non-pigmented mutant is unable to synthesize the red pigment prodigiosin, the redundant amino acids certainly have another destination. We have found 68 DEGs were enriched in 11 amino acid degradation pathways, and most of them were upregulated (Table 5). Therefore, the amino acids which were originally prepared for prodigiosin synthesis or other high-cost systems were probably degraded. Activated bacterial amino acid degradation pathways also suggested extra substrate for over-consuming. In this study, we have found at least five gene clusters related to the amino acid and peptide import pathways that were generally upregulated in the mutant SCQ1-3M (Table 6). The upregulated amino acid transport pathways provided potential routes for non-pigmented mutants to uptake extra amino acids. Taken together, the non-pigmented mutant shut down several high-cost systems and activated amino degradation and transport pathways to obtain extra nutrients, which contributed to bacterial growth.

## Conclusion

**Figure 7:**
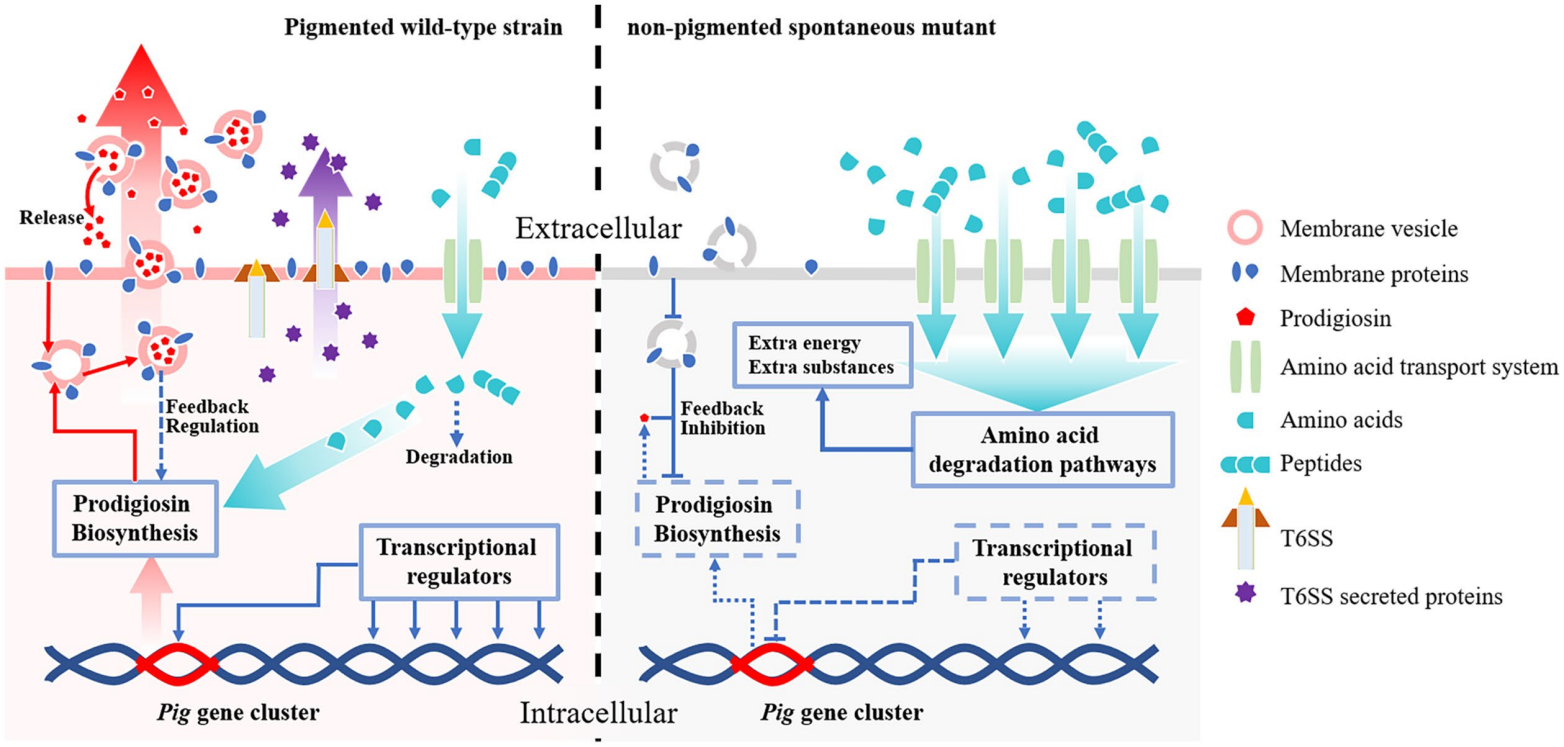
The potential mechanisms contribute to competitive growth advantage in *S. marcescens* spontaneous mutants under laboratory conditions. Both the wild-type strain and its spontaneous mutants preserve the complete prodigiosin synthesis gene cluster (*Pig* gene cluster). In the pigmented wild-type strain (left side), the *pig* genes are highly expressed under the control of transcriptional regulators, and the pigment is massively produced. The abundant prodigiosin is stored in membrane vesicles and secreted outside the cell by vesicular transport pathways to maintain lower intracellular concentrations which avoid feedback inhibition. The wild-type strain also consumes necessary resources to maintain some high-cost systems, e.g., T6SS. The amino acids required for prodigiosin synthesis and other systems are imported *via* amino acid transport systems, in which only a small amount of amino acids is degraded. In the non-pigmented spontaneous mutant (right side), the expression of the *pig* genes is highly suppressed by several factors. The transcriptional regulators, e.g., the positive regulator *slyA*, are down-expressed, which results in downregulated *pig* genes. Lack of essential membrane proteins for vesicle formation also potentially contribute to prodigiosin dyssynthesis. In this scenario, prodigiosin is neither correctly stored nor secreted outside the cells, which leads to strong feedback inhibition effects. Besides the pigment synthesis system, the non-pigmented mutant also shuts down T6SS or other high-cost systems to save resources. In addition, both amino acid transport- and degradation-pathways associated proteins are over-expressed in the non-pigmented mutant, which supplies extra nutrients and energies for bacterial growth. Therefore, the non-pigmented mutant shows a competitive growth advantage over the wild-type strain, which rapidly replaces the latter in laboratory conditions.

We use transcriptomic approaches to analyze the non-pigmented spontaneous mutant of *S. marcescens*, which rapidly replaces the wild-type parent strain within a few passages under laboratory conditions. Unexpectedly, the *Pig* gene cluster responsible for prodigiosin biosynthesis is completely preserved in the non-pigmented mutants, but significantly changed at the transcriptional level. In addition, qRT-PCR and growth analysis indicate that the downregulated *pig* genes are correlated with the growth advantage. The RNA-seq data suggests that the transcriptional regulators play important roles in prodigiosin dyssynthesis of the non-pigmented mutant SCQ1-3M. It is supported by deletion of the relative downstream transcriptional regulator *slyA*, in which the pigment production of Δ*slyA* mutant is greatly reduced because of the strongly repressed *pig* genes. However, the different bacterial growth patterns and *slyA* expression levels of different color morphotypes suggest other factors may contribute to bacterial growth. In SCQ1-3M, the transcriptomic analysis reveals that the high-cost systems, including membrane proteins and T6SS, are suppressed, which may affect prodigiosin synthesis indirectly according to previous reports. In contrast, both amino acid degradation and import pathways are activated, which provides extra substances for bacterial growth (Figure 7). Generally, *S. marcescens* shuts down high-cost systems (including prodigiosin biosynthesis) but activates amino acid metabolite and transport systems for extra resources which contribute to growth advantage under laboratory conditions.

## Data Availability Statement

The datasets generated for this study can be found in the National Center for Biotechnology Information (NCBI, https://www.ncbi.nlm.nih.gov/). The accession number of the *S. marcescens* SCQ1 chromosome is CP063354. Raw sequence read data for Genome-seq of SCQ1, SCQ1-1M, SCQ1-2M, SCQ1-3M, and SCQ1-4M has been deposited in the NCBI SRA with accessions SRR12825186, SRR12737688, SRR12825185, SRR12825184, and SRR12825183, respectively, under BioProject PRJNA386063. Raw sequence read data for RNA-seq has been deposited in the NCBI SRA with BioSample accessions from SAMN21893314 to SAMN21893319 under BioProject PRJNA767201.

## Author Contributions

TX, WZ, and YW designed the study and drafted the manuscript. TX, CX, JX, RL, and WZ performed the experiments and data analysis. NW, LX, YZ, ML, XM, and ZM edited the manuscript. All authors contributed to the article and approved the submitted version.

## Funding

This work was supported by China Agriculture Research System (No. CARS-22), the Fund of China Qujing Academician and Experts Workstation (grant number WYJ20170413), and the Fundamental Research Funds for the Central Universities (XDJK2020C047).

## Conflict of Interest

The authors declare that the research was conducted in the absence of any commercial or financial relationships that could be construed as a potential conflict of interest.

## Publisher’s Note

All claims expressed in this article are solely those of the authors and do not necessarily represent those of their affiliated organizations, or those of the publisher, the editors and the reviewers. Any product that may be evaluated in this article, or claim that may be made by its manufacturer, is not guaranteed or endorsed by the publisher.

## Footnotes

^1^ http://www.r-project.org/

^2^ https://crm.vazyme.com/cetool/singlefragment.html

^3^ https://www.ncbi.nlm.nih.gov/genome/browse/#!/prokaryotes/1112/

## Abbreviations

*Pig* gene cluster: Prodigiosin biosynthesis gene cluster
qRT-PCR: Quantitative real-time RT-PCR
FC: Fold changes
T6SS: Type VI secretion system
OMV: Outer membrane vesicle
