# Supplementary material for: Transcriptomic Analysis Reveals Competitive Growth Advantage of Non-pigmented Serratia marcescens Mutants
Source: Front Microbiol. 2022 Jan 4;12:793202. doi: 10.3389/fmicb.2021.793202 (PMC8764370; doi:10.3389/fmicb.2021.793202)
Supplement: Supplementary file 1 [file Data_Sheet_1.docx]

**Table S1. Primers used in this study.**

| **Primer name** | **Description** | **Primer sequence (5'→ 3')** | **Product size (bp)** |
| --- | --- | --- | --- |
| **For qRT-PCR of *pig* genes** | | | |
| rpoB-F | *rpoB* | CGAAGACGAGATGTACCCGG | 114 |
| rpoB-R |  | TCACCCAGATTCACACACGG |  |
| pigA-F | *pigA* | ATCAACCGCTCAACCCTGTG | 135 |
| pigA-R |  | GCGGACACGCCCATAAAG |  |
| pigB-F | *pigB* | CTGCTGATGATGGCGGGATT | 128 |
| pigB-R |  | GGCTTGGGTTTGCGGTGGT |  |
| pigC-F | *pigC* | AGTTCACGACCCGCTACGC | 130 |
| pigC-R |  | AGGTTATGCCTCTGCTGTTTCA |  |
| pigD-F | *pigD* | AACGCATTCTGTTCAATCGCAC | 201 |
| pigD-R |  | CGCCTTCATTGTTGTGTCCC |  |
| pigE-F | *pigE* | CGGGCTACGGGTGCCTAAAC | 120 |
| pigE-R |  | CAGTCTGTTCGGGAATGGAAATGTA |  |
| pigF-F | *pigF* | GCTTCATCGCTTCCGAAACAC | 107 |
| pigF-R |  | GCTCTCTATGCCGCCGTCG |  |
| pigG-F | *pigG* | GATTCCGCCTCCATTTTCG | 150 |
| pigG-R |  | CCCTGCGGCGACTTGC |  |
| pigH-F | *pigH* | GGGTTAGGCGGCTTCGTT | 159 |
| pigH-R |  | CTTGGTCAGTCGCTCGGGT |  |
| pigI-F | *pigI* | CGGCGACTGGGTGGAGAC | 127 |
| pigI-R |  | CGAGGGATGGTGATGAAGGAT |  |
| pigJ-F | *pigJ* | CAGCACCTACACCGCTCACG | 190 |
| pigJ-R |  | GTTGCTGGCGACGATAATGG |  |
| pigK-F | *pigK* | TGGGTGGAGACGACCGATT | 207 |
| pigK-R |  | AACGACTTCCGCCAACTGAT |  |
| pigL-F | *pigL* | CGAGGCGGAGCAGCGATT | 165 |
| pigL-R |  | CTGCTCGCCAAACGCGTGCCGT |  |
| pigM-F | *pigM* | CAAGTGGCGTTTCATCCTCG | 140 |
| pigM-R |  | TGCTCGGCACTTTCGTCGTT |  |
| pigN-F | *pigN* | GGCTGGCGGTGATGATGGT | 154 |
| pigN-R |  | TGATAATCGCTGCCGAAGTGG |  |
| **For qRT-PCR confirmation of RNA-seq** | | | |
| pho-F | SMR_GM002214 | GGCATCTGTCGGCGGTGAA | 146 |
| pho-R |  | GCGTCCCAGCAGGAGGTGTT |  |
| pigF-F | *pigF* | GCTTCATCGCTTCCGAAACAC | 107 |
| pigF-R |  | GCTCTCTATGCCGCCGTCG |  |
| BsmB-F | SMR_GM002048 | CGGCAACGCAGAAGTGAC | 159 |
| BsmB-R |  | GTGCTGCTGTTGATGGTGTAAT |  |
| pigH-F | *pigH* | GGGTTAGGCGGCTTCGTT | 159 |
| pigH-R |  | CTTGGTCAGTCGCTCGGGT |  |
| ompC-F | SMR_GM004458 | ACAACAAAGACGGTAATAAACTGG | 132 |
| ompC-R |  | CGCTGATTTGCGTTTCG |  |
| vgrG1-F | SMR_GM004066 | ACGCTGTGGTATCTGACGC | 136 |
| vgrG1-R |  | CCCAGATGCGGTAGTCGTAA |  |
| hcp1-F | SMR_GM004082 | GCACTGCTCCAGCGGTAA | 98 |
| hcp1-R |  | CGTCGTCCAGGGTGATTT |  |
| vgrG-F | SMR_GM004093 | CAACCGCTGACGCTGGAGAT | 85 |
| vgrG-R |  | TGACGGCGATGGTGGTGA |  |
| slyA-F | SMR_GM003301 | GAGCAGTCGCAAATCCAGTT | 181 |
| slyA-R |  | CGCTGTCCACTTCCCTGATG |  |
| tran-F | SMR_GM000582 | AAACCACCAACGCACAG | 167 |
| tran-R |  | CACGGTGGATTGCTGAAC |  |
| kdpE-F | SMR_GM002319 | CTGCGGGTGTTTGAAAGTGAA | 184 |
| kdpE-R |  | TGTCCTCTTCGGCGTTGC |  |
| paaB-F | SMR_GM004149 | CGCCGTAATGAAGGCTGCTC | 140 |
| paaB-R |  | CCGTCGGGAATGGTGTAAAA |  |
| astE-F | SMR_GM003928 | TGACGCTGGAAGGGAACGAA | 185 |
| astE-R |  | GCCGACCAGTTGATTGAGCAGTT |  |
| dthD-F | SMR_GM000015 | CTGGCGGTCAATCTGAAAGG | 122 |
| dthD-R |  | TTAGGCAAAGCCACCACCC |  |
| cpxP-F | SMR_GM001139 | TCTGCCTGGTGTCAATGTAGC | 94 |
| cpxP-R |  | TTCCGCCTGGGCATACA |  |
| dppB-F | SMR_GM001348 | GGACAAGCCGCTCTATCAACAA | 107 |
| dppB-R |  | GAACGAACTCGCTCCAGACG |  |
| livF-F | SMR_GM004266 | CGGGCAAGTCCACGCTGTT | 119 |
| livF-R |  | GCCGCTGCTGGCAACAAAGT |  |
| paaE-F | SMR_GM004152 | GCGGCGTTGCTATTCCATC | 120 |
| paaE-R |  | GCATCGCCGACTTTCAGCAT |  |
| malX-F | SMR_GM003342 | TGTTCGCCATCGCCATTC | 116 |
| malX-R |  | GGCGGTCAGGTAGAAGTTGG |  |
| mnmC-F | SMR_GM002918 | GAAGGGTATCGTGTCTCGGTGGT | 130 |
| mnmC-R |  | GCCACAACCGCAGCGAATAA |  |
| dppA-F | SMR_GM004627 | CCAATACGCTGGTCTACTGTTCC | 185 |
| dppA-R |  | CTTGCCGTCTTCGCTCACC |  |
| pecT-F | SMR_GM004504 | TAGAGCAACTGGTCGGCAAAG | 92 |
| pecT-R |  | CCTGGCATAACCGAGAAGC |  |
| oppA-F | SMR_GM003783 | CGGTGCCTTATCTGGTTGAA | 95 |
| oppA-R |  | CGGCAGCGTCCACTTCT |  |
| **For mutant construction** | | | |
| slyA-U-F |  | tgtggaatcccgggagagctcGCGGCGTGTTGCCGTTAT | 1153 |
| slyA-U-R |  | gaatactcatCGGCTTGAGCCGATGATCG |  |
| slyA-D-F |  | tggtaaTATCACCGAACTGCAGAACAAATAA | 1099 |
| slyA-D-R |  | aagcttatcgataccgtcgacTGGCACATCCCCAGGATCT |  |
| Amp-F |  | gctcaagccgATGAGTATTCAACATTTCCGTGTCG | 861 |
| Amp-R |  | gcagttcggtgataTTACCAATGCTTAATCAGTGAGGC |  |
| slyA-1F |  | TCGAGATGGTGCAGGTGGT | 1591 |
| slyA-1R |  | GGATAATACCGCGCCACATA |  |
| slyA-2F |  | GTGGACAACAAGCCAGGGAT | 1348 |
| slyA-2R |  | GCAAAGTGACCCAGTGCGT |  |
| slyA-3F |  | TTCCGTGTCGCCCTTATTC | 2141 |
| slyA-3R |  | CCGAGCTGCATCGAGAAAC |  |
| slyA-F | *slyA* | GGAGCAGTCGCAAATCCAG | 252 |
| slyA-R |  | AGCCCTACCAACAGATGCACT |  |
| **For complement construction** | | | |
| slyA-hb-F |  | ggtaccgggccccccctcgagTGAATCAGCAGCCATAAGACCA | 734 |
| slyA-hb-R |  | cgcggtggcggccgctctagaTTATTTGTTCTGCAGTTCGGTGA |  |
| M13F |  | TGTAAAACGACGGCCAGT | 989 |
| M13R |  | AGCGGATAACAATTTCACACAGGA |  |

**Table S3. The 3^th^ significantly enriched pathways with *q*-value < 0.05.**

| **Pathway** | **Pathway ID** | **K_ID** | **Gene_ID** | **log_2_(FC)** | **Description** |
| --- | --- | --- | --- | --- | --- |
| Prodigiosin biosynthesis | ko00333 | K21428 | SMR_GM002222 | -4.94 | pigD; 3-acetyloctanal synthase |
|  |  | K21778 | SMR_GM002221 | -6.06 | pigE; 3-acetyloctanal aminotransferase |
|  |  | K21779 | SMR_GM002224 | -14.91 | pigB; 2-methyl-3-n-amyl-dihydropyrrolel dehydrogenase |
|  |  | K21780 | SMR_GM002217 | -4.66 | pigI, redM; L-proline |
|  |  | K21782 | SMR_GM002225 | -8.22 | pigA, redW; L-prolyl-PCP dehydrogenase |
|  |  | K21783 | SMR_GM002216 | -3.28 | pigJ, redX; beta-ketoacyl ACP synthase |
|  |  | K21784 | SMR_GM002218 | -5.87 | pigH, redN; 4-hydroxy-2,2'-bipyrrole-5-methanol synthase |
|  |  | K21785 | SMR_GM002213 | -11.89 | pigM, redV; 4-hydroxy-2,2'-bipyrrole-5-methanol dehydrogenase |
|  |  | K21786 | SMR_GM002220 | -8.48 | pigF, redI; 4-hydroxy-2,2'-bipyrrole-5-carbaldehyde O-methyltransferase |
|  |  | K21787 | SMR_GM002223 | -6.40 | pigC, redH; prodigiosin/undecylprodigiosin synthetase |
| Biofilm formation | ko02025 | K01658 | SMR_GM003749 | -12.75 | trpG; anthranilate synthase component II |
|  |  | K11890 | SMR_GM004078 | -4.61 | impM; type VI secretion system protein ImpM |
|  |  | K11891 | SMR_GM004077 | -4.57 | impL, vasK, icmF; type VI secretion system protein ImpL |
|  |  | K11893 | SMR_GM004075 | -2.80 | impJ, vasE; type VI secretion system protein ImpJ |
|  |  | K11900 | SMR_GM004081 | -2.78 | impC; type VI secretion system protein ImpC |
|  |  | K11901 | SMR_GM004080 | -2.75 | impB; type VI secretion system protein ImpB |
|  |  | K11902 | SMR_GM004079 | -2.97 | impA; type VI secretion system protein ImpA |
|  |  | K11903 | SMR_GM000574 | -1.24 | hcp; type VI secretion system secreted protein Hcp |
|  |  | K11903 | SMR_GM004082 | -3.41 | hcp; type VI secretion system secreted protein Hcp |
|  |  | K11907 | SMR_GM004091 | -3.44 | vasG, clpV; type VI secretion system protein VasG |
| Arginine and proline metabolism | ko00330 | K00137 | SMR_GM000166 | 2.42 | prr; aminobutyraldehyde dehydrogenase |
|  |  | K00657 | SMR_GM000064 | -1.06 | speG, SAT; diamine N-acetyltransferase |
|  |  | K00673 | SMR_GM003931 | 1.89 | astA; arginine N-succinyltransferase |
|  |  | K00840 | SMR_GM003932 | 2.03 | astC; succinylornithine aminotransferase |
|  |  | K01484 | SMR_GM003929 | 2.63 | astB; succinylarginine dihydrolase |
|  |  | K01581 | SMR_GM001649 | 1.44 | E4.1.1.17, ODC1, speC, speF; ornithine decarboxylase |
|  |  | K05526 | SMR_GM003928 | 3.37 | astE; succinylglutamate desuccinylase |
|  |  | K06447 | SMR_GM003930 | 2.62 | astD; succinylglutamic semialdehyde dehydrogenase |
|  |  | K09470 | SMR_GM003150 | 1.76 | puuA; gamma-glutamylputrescine synthase |
|  |  | K09471 | SMR_GM001748 | 1.46 | puuB, ordL; gamma-glutamylputrescine oxidase |
|  |  | K12658 | SMR_GM002917 | 2.60 | lhpA; 4-hydroxyproline epimerase |
|  |  | K21061 | SMR_GM002918 | 2.19 | lhpB; D-hydroxyproline dehydrogenase subunit beta |
|  |  | K21062 | SMR_GM002915 | 3.00 | lhpC; 1-pyrroline-4-hydroxy-2-carboxylate deaminase |

**Table S4. DEGs involved in amino acid metabolism.**

| **Gene ID** | **Descriptions** | **log_2_(FC)** |
| --- | --- | --- |
| **Phenylalanine metabolism** | | |
| SMR_GM002908 | paaH, hbd, fadB, mmgB; 3-hydroxybutyryl-CoA dehydrogenase | -4.33 |
| SMR_GM004155 | paaH, hbd, fadB, mmgB; 3-hydroxybutyryl-CoA dehydrogenase | 1.90 |
| SMR_GM004162 | tyrB; aromatic-amino-acid transaminase | 2.78 |
| SMR_GM004153 | paaF, echA; enoyl-CoA hydratase | 2.79 |
| SMR_GM004158 | paaK; phenylacetate-CoA ligase | 2.34 |
| SMR_GM004148 | paaA; ring-1,2-phenylacetyl-CoA epoxidase subunit PaaA | 2.30 |
| SMR_GM004149 | paaB; ring-1,2-phenylacetyl-CoA epoxidase subunit PaaB | 3.00 |
| SMR_GM004152 | paaE; ring-1,2-phenylacetyl-CoA epoxidase subunit PaaE | 2.78 |
| SMR_GM004154 | paaG;2-(1,2-epoxy-1,2-dihydrophenyl) acetyl-CoA isomerase | 2.03 |
| **Arginine and proline metabolism** | | |
| SMR_GM000166 | prr; aminobutyraldehyde dehydrogenase | 2.42 |
| SMR_GM000064 | speG, SAT; diamine N-acetyltransferase | -1.06 |
| SMR_GM003931 | astA; arginine N-succinyltransferase | 1.90 |
| SMR_GM003932 | astC; succinylornithine aminotransferase | 2.03 |
| SMR_GM003929 | astB; succinylarginine dihydrolase | 2.63 |
| SMR_GM001649 | E4.1.1.17, ODC1, speC, speF; ornithine decarboxylase | 1.44 |
| SMR_GM003928 | astE; succinylglutamate desuccinylase | 3.37 |
| SMR_GM003930 | astD; succinylglutamic semialdehyde dehydrogenase | 2.62 |
| SMR_GM003150 | puuA; gamma-glutamylputrescine synthase | 1.76 |
| SMR_GM001748 | puuB, ordL; gamma-glutamylputrescine oxidase | 1.46 |
| SMR_GM002917 | lhpA; 4-hydroxyproline epimerase | 2.60 |
| SMR_GM002918 | lhpB; D-hydroxyproline dehydrogenase subunit beta | 2.193 |
| SMR_GM002915 | lhpC; 1-pyrroline-4-hydroxy-2-carboxylate deaminase | 3.00 |
| **Tryptophan metabolism** | | |
| SMR_GM004098 | TDO2, kynA; tryptophan 2,3-dioxygenase | 1.04 |
| SMR_GM002463 | ASMT; acetylserotonin O-methyltransferase | -1.47 |
| SMR_GM001724 | E2.3.1.9, atoB; acetyl-CoA C-acetyltransferase | 2.84 |
| SMR_GM004153 | paaF, echA; enoyl-CoA hydratase | 2.79 |
| SMR_GM004616 | ipdC; indolepyruvate decarboxylase | -1.26 |
| **Histidine metabolism** | | |
| SMR_GM002718 | hisG; ATP phosphoribosyltransferase | 1.32 |
| SMR_GM003162 | hutI, AMDHD1; imidazolonepropionase | -1.75 |
| SMR_GM003161 | hutG; formiminoglutamase | -2.29 |
| SMR_GM001938 | hutU, UROC1; urocanate hydratase | 1.002 |
| SMR_GM003164 | hutF; formimidoylglutamate deiminase | -1.68 |
| **beta-Alanine metabolism** | | |
| SMR_GM000166 | prr; aminobutyraldehyde dehydrogenase | 2.42 |
| SMR_GM001013 | mmsA, iolA, ALDH6A1; malonate-semialdehyde dehydrogenase (acetylating) / methylmalonate-semialdehyde dehydrogenase | 1.06 |
| SMR_GM000659 | puuE; 4-aminobutyrate aminotransferase | 1.18 |
| SMR_GM004153 | paaF, echA; enoyl-CoA hydratase | 2.79 |
| **Alanine, aspartate and glutamate metabolism** | | |
| SMR_GM003555 | GLUD1_2, gdhA; glutamate dehydrogenase (NAD(P)+) | 1.48 |
| SMR_GM001049 | E1.4.1.4, gdhA; glutamate dehydrogenase (NADP+) | 1.39 |
| SMR_GM000659 | puuE; 4-aminobutyrate aminotransferase | 1.18 |
| SMR_GM002813 | E3.5.1.1, ansA, ansB; L-asparaginase | 1.32 |
| SMR_GM003805 | E3.5.1.1, ansA, ansB; L-asparaginase | -1.37 |
| SMR_GM001543 | aspA; aspartate ammonia-lyase | 1.40 |
| SMR_GM001195 | glnA, GLUL; glutamine synthetase | 2.53 |
| **Valine, leucine and isoleucine degradation** | | |
| SMR_GM001013 | mmsA, iolA, ALDH6A1; malonate-semialdehyde dehydrogenase (acetylating) / methylmalonate-semialdehyde dehydrogenase | 1.06 |
| SMR_GM001724 | E2.3.1.9, atoB; acetyl-CoA C-acetyltransferase | 2.84 |
| SMR_GM001722 | E2.8.3.5A, scoA; 3-oxoacid CoA-transferase subunit A | 3.46 |
| SMR_GM004153 | paaF, echA; enoyl-CoA hydratase | 2.79 |
| **Tyrosine metabolism** | | |
| SMR_GM001764 | hpaE, hpcC; 5-carboxymethyl-2-hydroxymuconic-semialdehyde dehydrogenase | 1.39 |
| SMR_GM004162 | tyrB; aromatic-amino-acid transaminase | 2.78 |
| SMR_GM001767 | hpaH; 2-oxo-hept-3-ene-1,7-dioate hydratase | 1.46 |
| SMR_GM001768 | hpaI, hpcH; 4-hydroxy-2-oxoheptanedioate aldolase | 2.05 |
| SMR_GM003474 | adhP; alcohol dehydrogenase, propanol-preferring | -1.57 |
| **Lysine degradation** | | |
| SMR_GM001724 | E2.3.1.9, atoB; acetyl-CoA C-acetyltransferase | 2.84 |
| SMR_GM004153 | paaF, echA; enoyl-CoA hydratase | 2.79 |
| SMR_GM002912 | dpkA, lhpD; delta1-piperideine-2-carboxylate reductase | 2.44 |
| **Glycine, serine and threonine metabolism** | | |
| SMR_GM001153 | tdh; threonine 3-dehydrogenase | 1.51 |
| SMR_GM000483 | betB, gbsA; betaine-aldehyde dehydrogenase | 4.01 |
| SMR_GM000436 | GLDC, gcvP; glycine dehydrogenase | 1.06 |
| SMR_GM001154 | kbl, GCAT; glycine C-acetyltransferase | 1.36 |
| SMR_GM002747 | E4.3.1.17, sdaA, sdaB, tdcG; L-serine dehydratase | 2.79 |
| SMR_GM003896 | E4.3.1.17, sdaA, sdaB, tdcG; L-serine dehydratase | 1.10 |
| SMR_GM001264 | E4.3.1.19, ilvA, tdcB; threonine dehydratase | 1.43 |
| **Cysteine and methionine metabolism** | | |
| SMR_GM004162 | tyrB; aromatic-amino-acid transaminase | 2.78 |
| SMR_GM002526 | TST, MPST, sseA; thiosulfate/3-mercaptopyruvate sulfurtransferase | 1.06 |
| SMR_GM002437 | E3.3.1.1, ahcY; adenosylhomocysteinase | 1.73 |
| SMR_GM002747 | E4.3.1.17, sdaA, sdaB, tdcG; L-serine dehydratase | 2.79 |
| SMR_GM003896 | E4.3.1.17, sdaA, sdaB, tdcG; L-serine dehydratase | 1.10 |
| SMR_GM001935 | E4.4.1.11; methionine-gamma-lyase | -4.03 |





**Figure S1.** Quantitative RT-PCR (qRT-PCR) analysis of DEGs selected in RNA-Seq analysis of SCQ1-3M *vs* SCQ1. The −ΔΔCt is used to determine the fold change in mRNA level using *rpoB* as internal control and the data are displayed as mean ± SE (n=3). *pho*: 4'-phosphopantetheinyl transferase, *pigF*: O-methyltransferase, *BsmB*: BsmB family protein BsmB, *pigH*: 7-keto-8-aminopelargonate synthetase, *ompC*: Porin OmpC, *vgrG1*: ImpA family type VI secretion-associated protein, *hcp1*: type VI secretion system effector, Hcp1 family, *vgrG*: type IV secretion protein Rhs, *slyA*: transcriptional regulator slyA, *tran*: transcriptional regulator, *kdpE*: KDP operon response regulator KdpE, *paaB*: phenylacetate-CoA oxygenase subunit PaaB, *astE*: succinylglutamate desuccinylase, *dthD*: D-threitol dehydrogenase, *cpxP*: periplasmic protein CpxP, *dppB*: binding--dependent transport system inner membrane component family protein, *livF*: phosphonate-transporting ATPase, *paaE*: phenylacetic acid degradation protein, *malX*: PTS system maltose and glucose-specific IICB components, *mnmC*: D-amino-acid oxidase, dppA: ABC transporter substrate-binding protein, *pecT*: transcriptional regulator LrhA, *oppA*: periplasmic substrate-binding component of an ABC superfamily oligopeptide transporter.
